# Where, when, how, and with whom do young university students engage in physical activity? A cluster-derived spectrum of patterns across 16 European universities

**DOI:** 10.1371/journal.pone.0360751

**Published:** 2026-11-04

**Authors:** Clément Llena, Louise Fermon, Søren Andkjær, Audran Aulanier, Félix Berrigan, Nico Bortoletto, Mário Jorge Costa, Yvonne Delevoye-Turrell, Thibaut Derigny, Olivier Dieu, Murielle Garcin, Joseph Gandrieau, Ricardo Jorge Fernandes, Libor Flemr, Christopher Fullerton, Hee Jung Hong, Aurore Huchez, Isabelle Joing, Vanessa Lentillon-Kaestner, Alexis Mauger, Barbara Mazza, Léa Mekkaoui, Enrico Michelini, Annette Michelsen la Cour, Alexandre Mouton, Przemysław Nosal, Pedro R. Olivares, Bjørn Harald Olstad, Marek Rejman, Susana Soares, João Paulo Vilas-Boas, Tobias Vogt, Alessandro Porrovecchio, François Potdevin

**Affiliations:** 1 Univ. Lille, Univ. Artois, Univ. Littoral Côte d’Opale, ULR 7369 – URePSSS – Unité de Recherche Pluridisciplinaire Sport Santé Société, Lille, France; 2 Université de Sherbrooke, Québec, Canada; 3 University of Southern Denmark, Odense, Denmark; 4 TREE UMR 6031, Université de Pau et des Pays de l’Adour – CEMS/EHESS, Paris, France; 5 University of Teramo, Teramo, Italy; 6 Department of Sport Sciences, Exercise and Health, University of Trás-os-Montes and Alto Douro, Vila Real, Portugal; 7 Univ. Lille, CNRS, UMR 9193 – SCALab – Sciences Cognitives et Sciences Affectives, Lille, France; 8 Université de Pau et des Pays de l’Adour, E2S UPPA, MEPS, Anglet, France; 9 Univ. Littoral Côte d’Opale, Univ. Artois, Univ. Lille, ULR 7369 – URePSSS – Unité de Recherche Pluridisciplinaire Sport Santé Société, Dunkerque, France; 10 Université Côte d’Azur, Laboratoire Motricité Humaine Expertise Sport Santé (LAMHESS), Nice, France; 11 CIFI2D and LABIOMEP, Faculty of Sport, University of Porto, Porto, Portugal; 12 College of Physical Education and Sport PALESTRA, Prague, Czechia; 13 Faculty of Health, Sport and Society, University of Stirling, Stirling, Scotland; 14 Haute école pédagogique du canton de Vaud, Lausanne, Switzerland; 15 University of Kent, London and South East University Group, Canterbury, England; 16 Sapienza University of Rome, Rome, Italy; 17 University of Osnabrück, Jahnstr. 75, 49080 Osnabrück, Germany; 18 University of Liège, RUCHE – Research Unit for a life-Course perspective on Health & Education, Liège, Belgium; 19 Adam Mickiewicz University, Poznań, Poland; 20 University of Huelva, Huelva, Spain; 21 Norwegian School of Sport Sciences, Oslo, Norway; 22 University of Health and Sport Sciences, Wrocław, Poland; 23 German Sport University Cologne, Cologne, Germany; Universidad Santiago de Cali, COLOMBIA

## Abstract

Young adults are particularly at risk of dropping out of physical activity (PA). European policies have addressed this issue by promoting PA and encouraging better monitoring of PA through standardized and specific measures of its quantity. The current paper used an ecological approach that considers interrelated determinants at the individual, social, environmental, and policy levels to survey 2,165 undergraduate students (65.3% female) aged 18–25 years from 16 universities via an online questionnaire as part of the “How Young People in Europe Move” (HYPEM) project. A cluster analysis of mixed data was performed to identify profiles of sport and PA participation among undergraduates from 12 European countries and to examine their associations with individual and contextual characteristics. The results showed that individual and contextual characteristics were associated with spatial, temporal, social, intensity-related, and equipment-related dimensions of PA participation. Additionally, space, time and equipment were key variables for profiling students. Furthermore, the cluster analysis revealed three typologies of student sports and PA participation: ‘Autonomous’, ‘Networked Supervised’, and ‘Regularly Supervised’. These profiles suggest that undergraduate students engage in PA through various formats, ranging from flexible, self-organized practices to more structured, supervised forms of participation. Differences were also observed across participating institutions, indicating that contextual factors should be considered when interpreting these exploratory profiles. These profiles may help generate hypotheses about how university PA opportunities could be designed and evaluated in future prospective and intervention studies.

## Introduction

Physical activity (PA) provides health benefits to all populations [1]. However, it is widely recognized that the global population does not engage in sufficient PA, particularly at the moderate-to-vigorous intensities that have been shown to provide the greatest health benefits [2,3]. In Europe, 35% of citizens reported never participating in PA programs [4]. This increasing sedentary behavior is of concern as it leads to numerous negative health outcomes [5,6].

Adolescents and young adults are among the most studied populations when it comes to physical inactivity [2]. Several national reports [7] have shown that, on average, more than 50% of young people (aged 16–25) are not considered ‘active’. This transitional period, from adolescence to emerging adulthood, is particularly vulnerable to declines in PA participation [8]. Undergraduate students represent a subgroup of this population, undergoing a significant transitional period characterized by increased autonomy, but also an environment conducive to sedentary behaviors [2,9]. While university life is often liberating, it is also characterized by increased academic pressure and a more sedentary lifestyle, which may hinder regular PA participation. Indeed, although universities provide an environment conducive to promoting PA, numerous studies have shown that PA levels decline in this age group, particularly due to academic demands and time management challenges, which limit students’ opportunities to engage in regular PA [8,10].

Moreover, undergraduate students also encounter distinctive barriers during the transition to adulthood, such as the challenge of balancing academic responsibilities with leisure time, constraints in accessing sport facilities due to financial limitations, and a lack of awareness regarding the long-term health benefits of regular PA [11]. This transitional period, characterized by new life choices and responsibilities, is of paramount importance for the establishment of active lifestyle habits and the prevention of long-term sedentary behavior [10]. In this context, describing students’ PA and sport participation patterns and the factors associated with them may help formulate questions for future research on university PA promotion.

The adoption of an ecological model that incorporates multiple theories is critical to understanding the dynamics that influence university students’ engagement with PA. This model provides a comprehensive framework that considers interrelated determinants at the individual, social, environmental and policy levels [12,13]. In the present study, this ecological framework was employed both as a theoretical lens and as a means of organizing the variables included in the analysis. The individual-level factors included sex, age, field of study, employment status, and previous PA-related background. Social-level factors referred to the relational context of PA participation, such as practicing alone, with peers, in supervised settings, or with an animal. Environmental and organizational factors included the place of practice, timing, seasonality, duration, type of structure and equipment used. Finally, university and country indicators were considered as markers of the broader institutional and regional environments, although policy-level determinants were not measured directly.

In addressing these concerns, the present study advances beyond the conventional emphasis on the energetic dimension of PA [14] by adopting a broader definition, as proposed by Piggin (2020), which sees PA as “*people moving, acting and performing within culturally specific spaces and contexts, and influenced by a unique set of interests, emotions, ideas, instructions and relationships”* [15, p. 5]. In this study, PA is conceptualized as a multidimensional phenomenon that includes energy expenditure as well as contextual, social, and experiential dimensions of participation. However, it is empirically operationalized based on observable behavioral and contextual indicators, such as autonomy, supervision, location, timing, social context, intensity, and equipment. This definition encompasses not only sports activities but also different forms of PA that can occur in organized and non-organized contexts. These forms of PA aim to express, maintain or improve physical and/or mental health [16]. Given the European scope of the analysis, this study adopts the definitions of sport and PA from the European Sport Charter [17]. The Charter defines sport as ‘*all forms of physical activity which, through casual or organized participation, aim at expressing or improving physical fitness and mental well-being, forming social relationships, or achieving results in competition at all levels*’ (n.p.). In this study, PA refers to sport participation and PA participation (recreational or non-sport-related activities, such as cycling for transport, dancing, gardening, and walking [4,7]).

From a macro perspective, examining the broader dynamics impacting individuals’ engagement or disengagement with PA, a multifaceted ecological model has been developed [12,13]. The ecological approach uses a comprehensive framework to explain the whole of PA and proposes the consideration of interconnected determinants at all levels: individual, social, environmental and policy. From this perspective, the analysis of undergraduate students’ PA requires an understanding of the places, times, and social relationships they seek, as well as the forms of PA and sport they prefer.

Recent studies have indicated a shift in the patterns of PA and sports engagement among young people, with a tendency towards unstructured activities within the domestic environment [4,18]. A regional effect on the amount of PA and sports participation of the population has been identified in Europe, reinforcing the idea of “situated*”* differences in PA and sports favored by young adults [19]. For instance, the amount of PA and sports decreases from North to South and from West to East [20]. Most studies have examined cross-country disparities in PA levels [21]. Regional differences in PA and sport participation have also been identified at the European level, which supports the notion of a “location” effect on PA preferences among young adults [21]. While often framed as a national issue, some institutions advocate for regional and local strategies to support sustainable PA promotion and account for contextual differences [22].

Understanding how these factors are associated with students’ PA and sport behaviours may help formulate questions for future research. Examining students’ reported locations, timing, social contexts, and forms of PA can provide a broader description of participation patterns and inform the design of prospective and intervention studies.

While PA is often studied from a broad perspective, such as through the SLOTH model, which categorizes activity by Sleep, Leisure, Occupation, Transportation, and Home settings [23], or from an intensity-based “caloric” approach using light, moderate, and vigorous levels [24], less research has specifically examined how engagement in PA varies according to contextual factors such as location, timing, social interactions, and intensity across different PA and sport settings.

By conceptualizing engagement in PA as a phenomenon emerging from a network of interacting factors related to individual characteristics, the living environment, and the broader socio-environmental context [25,26], this study seeks to explore undergraduate students’ preferences for PA and sport participation across 12 European countries. Specifically, it examines how individual and contextual characteristics are associated with PA and sport participation across spatial, temporal, social, intensity, and equipment dimensions [18]. By identifying the most salient patterns of PA participation, this analysis may generate hypotheses about how university PA opportunities could be designed and evaluated in future research.

## Materials and methods

### Participants

Participants were recruited through convenience sampling at partner universities. Of the 4,321 individuals who initiated the survey, 1,061 did not provide questionnaire responses, and the remaining 3,260 underwent eligibility and data-completeness screening. Of these, 2,165 participants, including 1,413 women, were retained in the final analytic sample following the screening described in the Analysis section. Eligible participants were enrolled at a participating university, were aged 18–25 years, confirmed the truthfulness of their responses, and reported no health condition preventing participation in PA. Their mean age was 20.02 ± 1.45 years. Detailed participant characteristics are presented in Table 1.

**Table 1:** Characteristics of the participants.

*Sex*	
Women	1413(65.3%)
Men	752(34.7%)
***Field of study***	
Law and political science	120(5.5%)
Business and management	87(4.0%)
Medicine	298(13.8%)
Sports science	277(12.8%)
Humanities and social sciences or arts	458(21.2%)
Technology and engineering	272(12.6%)
Other field	653(30.1%)
***Employment***	
Yes	583(26.9%)
No	1582(73.1%)
Age^#^ (Years)	20.02 ± 1.45

Results are expressed as the number of respondents (in %).

#: data are in mean ± standard deviation.

The study’s participants were students from 12 countries across Europe, selected to ensure a geographically diverse sample. Each member of the university agreed to assist in the dissemination of the survey to their students, thus facilitating the recruitment process. This criterion made sense from an organizational point of view, as it allowed the researchers to reach a diverse pool of students while ensuring consistency in the context of university education. Details of the participating institutions are given in Table 2.

**Table 2:** Description of respondents included in the study, by country.

University (by country)	Number of respondents
University of Prague (Czech Republic)	521(24.1%)
Haute école pédagogique du canton de Vaud, Lausanne (Switzerland)	58(2.7%)
Norwegian School of Sport Sciences, Oslo (Norway)	51(2.4%)
German Sport University Cologne & Technical University of Dortmund (Germany)	106(4.9%)
University of Huelva (Spain)	65(3.0%)
University of Kent, Canterbury & University of Stirling (United Kingdom)	83(3.8%)
University of Liège (Belgium)	217(10.0%)
University of Lille (France)	184(8.5%)
University of Porto (Portugal)	125(5.7%)
University of Southern Denmark, Odense (Denmark)	54(2.5%)
University of Health and Sport Sciences, Wrocław & Adam Mickiewicz University, Poznań (Poland)	185(8.5%)
Sapienza University of Rome & University of Teramo (Italy)	516(23.9%)

### Study design

This study was conducted within the framework of a European research project (HYPEM) involving 16 partner institutions. A cross-sectional design was employed to collect data from undergraduate students across Europe at a single point in time. This methodological approach enabled the study to examine participants’ PA preferences and behaviors in the context of their university experience, obviating the necessity for long-term follow-up.

### Data collection procedure

A structured questionnaire was developed to collect data, encompassing five sections: (1) identification of preferred PA and sports participation; (2) identification of preferred types of PA practice; (3) general individual characteristics; (4) individual characteristics related to PA background; and (5) individual characteristics related to motives to participate in PA. Parts 1, 3, and 4 were adapted from French national surveys [27]. Part 2 was developed specifically for the present study. It investigates all physical and sporting activities across the dimensions of time, space, intensity, social relationships and equipment.

The French version of the questionnaire was translated into 10 languages (Czech, Danish, Dutch, English, German, Italian, Norwegian, Polish, Portuguese, and Spanish) to match the national languages of the different countries. Translations followed the parallel back-translation method (Brislin, 1986). This process involved bilingual experts who independently translated and back-translated the questionnaire. Subsequently, a review committee ensured the final version’s accuracy and cultural appropriateness [28].

Data collection occurred between September 2021 and January 2023. Each member directly invited students to a multilingual website where the questionnaire was hosted using LimeSurvey. The recruitment strategy was consistent across all universities, with the study being promoted through their established communication channels. However, the number of participants varied between institutions due to differences in institutional support, student engagement and availability. The study protocol was reviewed and approved by the Ethics Committee for Experimental Human Sciences at the University of Lille in France (CER-Lille; approval number 2021-475-S91) corresponding to the HYPEM project. It was conducted as a multi-site European study, coordinated by the University of Lille. This central ethics approval covered the overall research protocol, including the online questionnaire, consent procedure and data management plan. As recruitment and dissemination were conducted through partner universities, each institution was responsible for ensuring compliance with its own local institutional procedures and applicable national or regional regulations before distributing the questionnaire to students.

Prior to completing the questionnaire, participants were provided with information regarding the study’s objectives, the voluntary nature of their participation, the data protection procedures in place, and their rights as participants. Electronic informed consent was obtained prior to questionnaire completion.

### Analysis

Before the multivariate analyses, 3,260 individuals who provided questionnaire data were screened sequentially for eligibility and data completeness. Individuals were excluded because of missing age (n = 890), age outside the eligible range of 18–25 years (n = 80), missing year-of-study information (n = 1), or missing information on their participating university (n = 124). No individuals were excluded because of failure to confirm response truthfulness, a reported health condition preventing participation in PA, inconsistent responses, or statistical outlier screening (n = 0 for each). The final analytic sample comprised 2,165 participants. All values were checked against the predefined response options, and no multivariate outlier-detection procedure was applied to the mixed dataset.

To handle the mixed structure of the dataset, Factor Analysis of Mixed Data (FAMD) was conducted using the FactoMineR package in R [29], which allows continuous, categorical and ordinal variables to be analyzed within a single analytical framework. This analytical strategy aligns with previous applications of FAMD and cluster analysis in studies examining the PA participation profiles of female university students [30]. Continuous variables were standardized and categorical variables were encoded using a complete disjunctive indicator coding scheme. Ordinal variables were treated according to their ordered response scales. The FAMD procedure automatically balances the contribution of different variable types, thereby reducing the risk of one type of variable dominating the analysis. The resulting principal dimensions were then used as input for the clustering procedure.

First, hierarchical agglomerative clustering was performed to identify natural groupings in the data. The number of clusters was determined by examining the hierarchical clustering dendrogram, changes in within-cluster inertia, cluster size and the interpretability of alternative solutions. Based on these criteria, a three-cluster solution was retained. To further assess the robustness of the final solution, internal validation indices were computed for alternative solutions with two to six clusters. The average silhouette width and gap statistics were examined alongside cluster size and substantive interpretability. While these indices did not identify a single statistical optimum, the three-cluster solution offered the best balance between internal validation indices, parsimony, cluster size and interpretability. These validation indices are reported in S1 File. The final solution was then consolidated using the K-means algorithm, which refines cluster membership by minimising within-cluster variance.

Cluster interpretation was based on comparisons between cluster-specific means or proportions of categorical variables and the corresponding values in the overall sample. The V-test statistic was used to identify variables that were significantly over- or under-represented in each cluster based on their deviation from the overall sample distribution [31]. Associations between cluster membership and categorical supplementary variables were additionally examined using Pearson’s chi-square tests, with Cramér’s V reported as a measure of association strength. Descriptive statistics included means, standard deviations, frequencies, and proportions, as appropriate. The adequacy of the sample for factor analysis was confirmed using the Kaiser–Meyer–Olkin (KMO) index, which indicated good suitability (KMO = 0.849). All analyses were conducted in R version 4.3.0 using packages including FactoMineR, factoextra, cluster, psych, rstatix, and effectsize.

## Results

The obtained data are presented in Tables 3, 4 and 5, which illustrate three profiles of participants in PA and sports, according to their preferences in terms of spatial, temporal, social, intensity and equipment dimensions.

**Table 3:** Significant correlations between variables and dimensions identified through factorial analysis of mixed data (FAMD).

Dimension 1		Dimension 2	
*Positive correlation*		*Positive correlation*	
Supervised practice	0.7	Running/walking	0.59
Structured association	0.69	During holidays	0.57
During autumn	0.67	Use of ICT	0.56
During winter	0.66	Outdoors	0.54
During spring	0.65	During summer	0.52
Regular throughout the year	0.62	Weekends	0.47
Precise timetable	0.62	Autonomy	0.44
Closed facilities	0.58	Cycling	0.40
***Negative correlation***		***Negative correlation***	
Completely free	−0.56	Closed facilities	−0.25
Practice at home	−0.44	Precise timetable	−0.20
Less than 30 minutes	−0.44	Private structure	−0.18
Variable throughout the year	−0.42	Registration process	−0.18
With an animal	−0.42	Supervised practice	−0.16
Free timetable	−0.34	Club structure	−0.007
Internet program	−0.28	Less than 120 min	−0.006
Use of ICT	−0.22	Prescribed program	−0.006

Full variable definitions, response scales and coding decisions are provided in S2 File.

**Table 4:** Characteristics for each cluster of qualitative data (in %).

	Cluster 1 Autonomous 42.6%	Cluster 2 Networked Supervised 28.6%	Cluster 3 Regularly Supervised 28.8%
Sex*			
Men	35.1%	39.1% ^*c*^	25.8% ^*c*^
Women	51.3% ^*a,b*^	25.1% ^*a*^	23.6% ^*b*^
Field of study*			
Humanities, social sciences or arts	63.7% ^*a,b*^	22.1% ^*a*^	14.2% ^*b*^
Engineering and technology	43.0% ^*a*^	23.2% ^*a,c*^	33.8% ^*c*^
Sports Science	16.6% ^*a*^	68.6%* ^*a,c*^	14.8% ^*c*^
Student job*			
Yes	39.3% ^*a*^	41.4% ^*c*^	19.3% ^*a,c*^
None	48.1% ^*a,b*^	25.7% ^*a*^	26.2% ^*b*^
University* (only universities with significant results are shown here) ^¶^			
University of Teramo	31.5% ^*a*^	11.5% ^*a,c*^	57.0% ^*c*^
Sapienza University of Rome	32.8% ^*a*^	13.0% ^*a,c*^	54.2%* ^*c*^
University of Prague	56.5% ^*a*^	36.1% ^*c*^	7.4% ^*a,c*^
Adam Mickiewicz University, Poznań	71.4% ^*a,b*^	23.8% ^*a,c*^	4.8% ^*b,c*^
University of Health and Sport Sciences, Wrocław	44.3% ^*a*^	38.1% ^*c*^	17.6% ^*a,c*^
Technical University of Dortmund	10.0% ^*a*^	70.0% ^*a,c*^	20.0% ^*c*^
Norwegian School of Sport Sciences, Oslo	6.6% ^*a*^	86.6% ^*a,c*^	6.8% ^*c*^
University of Porto	8.0% ^*a*^	36.0% ^*a,c*^	56.0% ^*c*^
University of Lille	11.1% ^*a*^	57.7% ^*a,c*^	31.2% ^*c*^
University of Liège	7.2% ^*a*^	55.1% ^*a,c*^	37.7% ^*c*^

Full variable definitions, response scales and coding decisions are provided in S2 File.

Percentages are calculated within each category and indicate the proportion of participants in that category assigned to each cluster.

* Significant data (all for p < 0.05);

¶Only institutions that are significantly associated with cluster membership are displayed. Due to the unequal distribution of respondents across universities, these results should be interpreted as exploratory site-level associations.

^a^, ^b^ and ^c^ showed respectively significant differences between the Autonomous and the Networked Supervised, the Autonomous and the Regularly Supervised, and the Networked Supervised and the Regularly Supervised.

**Table 5:** Characteristics for each cluster of quantitative data (ordinal scale from 1 to 5).

	Cluster 1 *Autonomous* *42.6%*	Cluster 2 *Networked Supervised* *28.6%*	Cluster 3 *Regularly Supervised* *28.8%*	Mean ± SD
The different practices*				
Independent practice	2.19 ± 1.29 ^*b*^	2.13 ± 1.22 ^*c*^	0.97 ± 1.09^*b,c*^	1.87 ± 1.33
Supervised practice	0.35 ± 0.45 ^*a,b*^	1.20 ± 0.84 ^*a*^	1.56 ± 0.84 ^*b*^	0.89 ± 0.86
Practice indoors	1.09 ± 1.03 ^*a,b*^	2.14 ± 1.16 ^*a*^	2.93 ± 1.21 ^*b*^	1.85 ± 1.35
Practice at home	2.53 ± 1.26 ^*b*^	2.04 ± 1.01	1.04 ± 1.01 ^*b*^	1.76 ± 0.89
Outdoor activities	1.76 ± 1.27 ^*b*^	2.10 ± 1.12 ^*c*^	0.77 ± 0.89 ^*b,c*^	1.61 ± 1.25
With an animal	2.53 ± 1.19 ^*b*^	1.93 ± 1.18	1.21 ± 1.21 ^*b*^	2.03 ± 1.30
Practical periods*				
In autumn	2.20 ± 1.05 ^*b*^	3.40 ± 0.83	3.52 ± 0.79 ^*b*^	2.87 ± 1.12
In summer	2.43 ± 1.06 ^*a*^	3.5 ± 0.78 ^*a,c*^	2.90 ± 1.21 ^*c*^	2.79 ± 1.14
In winter	1.85 ± 1.13 ^*b*^	3.14 ± 1.03	3.43 ± 0.88 ^*b*^	2.61 ± 1.27
In spring	2.40 ± 1.00 ^*b*^	3.49 ± 0.72	3.54 ± 0.76 ^*b*^	3.00 ± 1.03
During the week	1.91 ± 1.13 ^*b*^	3.04 ± 1.06	3.01 ± 1.14 ^*b*^	2.51 ± 1.25
On weekends	1.72 ± 0.99 ^*a*^	2.92 ± 1.02 ^*a,c*^	1.84 ± 1.28 ^*c*^	2.10 ± 1.20
During holidays	2.20 ± 1.06	3.25 ± 0.93 ^*c*^	1.97 ± 1.14 ^*c*^	2.45 ± 1.17
At anytime	2.58 ± 1.1 ^*b*^	1.60 ± 1.05	0.93 ± 1.01 ^*b*^	1.8 ± 1.2
Regular throughout the year	1.94 ± 1.11 ^*b*^	3.19 ± 0.91	3.07 ± 0.96 ^*b*^	2.58 ± 1.18
Variable throughout the year	2.88 ± 0.97 ^*b*^	2.11 ± 1.29	1.85 ± 1.34 ^*b*^	2.40 ± 1.25
Physical activities*				
Running/walking	2.34 ± 1.17	2.95 ± 0.90 ^*c*^	1.44 ± 1.14 ^*c*^	2.32 ± 1.22
Cycling	0.92 ± 0.91 ^*a*^	1.48 ± 1.05 ^*a*^	0.61 ± 0.76	1.01 ± 0.97
Athletics	0.19 ± 0.51 ^*a*^	1.14 ± 1.19 ^*a,c*^	0.24 ± 0.70 ^*c*^	0.47 ± 0.91
Water sports	0.79 ± 0.72 ^*a*^	1.37 ± 0.96 ^*a,c*^	0.82 ± 1.01 ^*c*^	0.96 ± 0.91
Use of ICT*				
ICT Communication	0.60 ± 0.63 ^*a*^	1.15 ± 0.88 ^*a,c*^	0.51 ± 0.59 ^*c*^	0.73 ± 0.75
Functional ICT	0.94 ± 0.79 ^*a*^	1.60 ± 0.95 ^*a,c*^	0.57 ± 0.64 ^*c*^	1.02 ± 0.89
Duration of exercises*				
Less than 30 min	1.12 ± 0.97 ^*a,b*^	0.67 ± 0.76 ^*a*^	0.27 ± 0.53 ^*b*^	0.78 ± 0.89
Less than 60 min	2.10 ± 1.03 ^*a*^	1.82 ± 0.95 ^*a,c*^	0.85 ± 0.99 ^*c*^	1.66 ± 1.15
More than 60 min	1.54 ± 1.09 ^*a*^	2.61 ± 0.97 ^*a*^	2.89 ± 1.11	2.19 ± 1.23
More than 120 min	0.57 ± 0.72 ^*a,b*^	1.37 ± 1.01 ^*a*^	1.12 ± 1.11 ^*b*^	0.94 ± 0.98

The results are presented as the mean ± standard deviation.

Higher values indicate stronger endorsement of, or higher reported involvement in, the corresponding PA format.

Full variable definitions, response scales and coding decisions are provided in S2 File.

*Significant at p < .05;

^a^, ^b^ and ^c^ showed respectively significant differences between the Autonomous and the Networked Supervised, the Autonomous and the Regularly Supervised, and the Networked Supervised and the Regularly Supervised.

### Space, time and equipment: key variables for profiling students

The variables are organized in a two-dimensional plane. The variability among the variables is explained by 14.20% for dimension 1 and 7.18% for dimension 2. These two dimensions collectively account for 21.38% of the total variability. Table 3 presents the significant correlations between the variables and dimensions that were identified through the factorial analysis of the mixed data.

Concerning the first dimension, the variables that are most directly related are those that involve supervised practice, which is structured within clubs and practiced regularly during the academic year. Conversely, the variables that are inversely related to the first dimension are those that involve a complete absence of cost, practice occurring within the home environment, variability in the duration of the sport’s engagement over the course of the year, and a limited time commitment. Dimension 2, on the other hand, is associated with independent practice (particularly walking, running, and cycling), outdoor practice (during summer and weekends), and the overrepresentation of students utilizing ICT (Information and Communication Technologies). Conversely, practices with an inverse relationship include facilities in enclosed spaces and private structures, as well as specific times during supervised practices that require registration.

### Three types of clusters: Autonomous, networked supervised and regularly supervised

Fig 1 illustrates the clustering model, which identifies three distinct types of clusters. Cluster 1, labelled ‘Autonomous’ and comprising 42.6% of the participants, is predominantly characterized by engagement in autonomous PA. In contrast, Cluster 2, labelled ‘Networked Supervised’ (28.6% of the participants), and Cluster 3, labelled ‘Regularly Supervised’ (28.8% of participants), are primarily associated with structured forms of PA and sports. The characteristic variables for each cluster are detailed in Tables 4 and 5.

**Fig 1:**
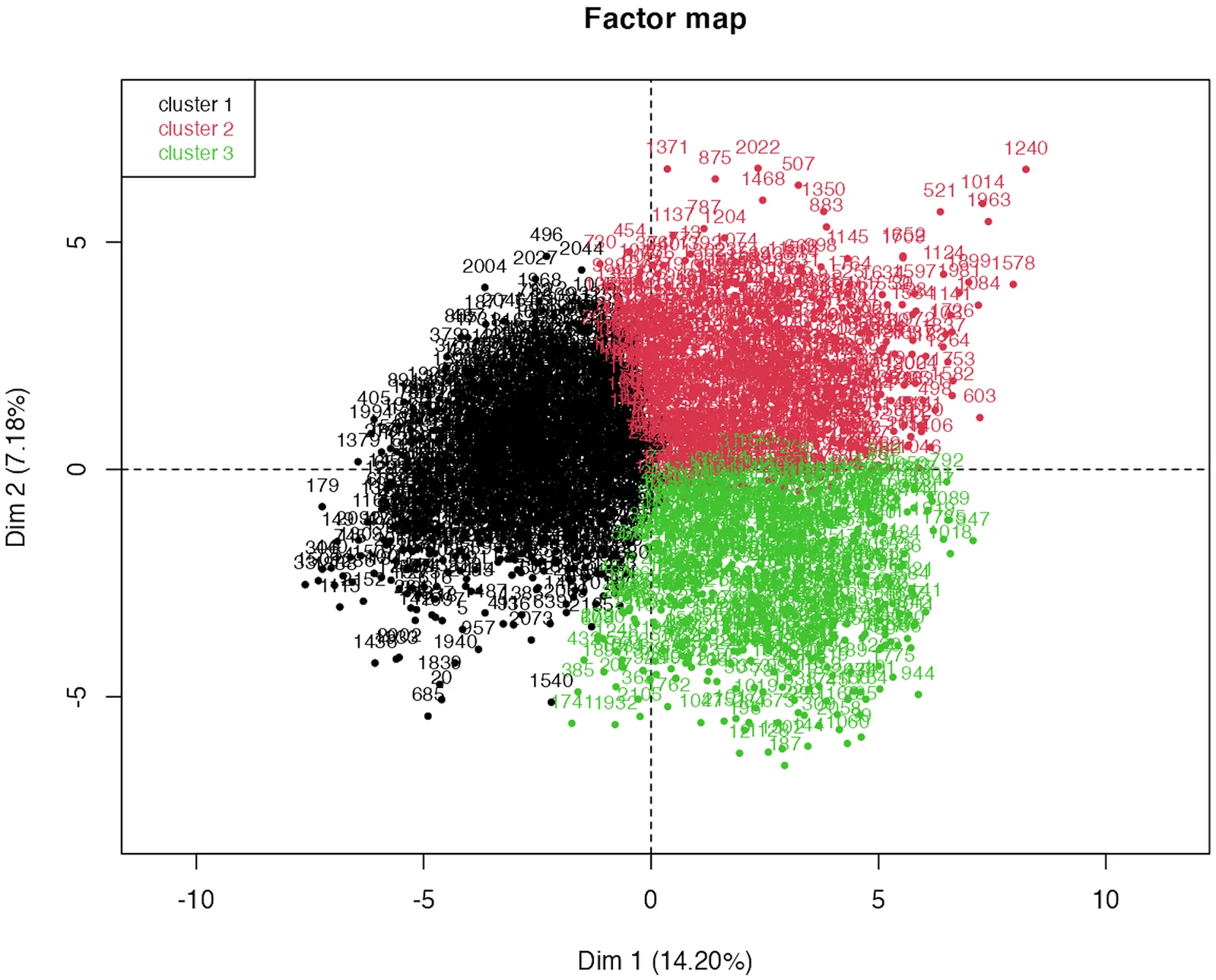
Two-dimensional factorial design distribution of the three clusters.

Cluster 1 (in black in Fig 1) is characterized by a population that practices independently (p < .05), outside university hours, free of charge (p < .05), often at home, with flexible schedules and highly variable frequency throughout the year (p < .05). A significant over-representation of students from humanities, social sciences, or arts (63.7%, p < .01) and women (51.3%, p < .001) was observed in this cluster. The highest proportions of students in this cluster were observed in the participating institutions located in Poznań (71.4%, p < .001), Prague (56.5%, p < .001), and Wrocław (44.3%, p < .001). Furthermore, this cluster included a higher proportion of unemployed students (48.1%, p < .001). The most prevalent activities among these students were walking alone (2.34 ± 1.17, p < .001) or with a pet (2.53 ± 1.19, p < .001).

Clusters 2 (in red in Fig 1) and 3 (in green in Fig 1) are characterized by students engaging in supervised and structured PA during the school day.

Cluster 2 consists predominantly of male students (39.1%; p < .001), many of whom hold a job (40%; p < .001) and participate in group-based activities, such as club sports, throughout the year (p < .05). The participating institutions with the highest representation in this cluster included Oslo (86.6%), Dortmund (70.0%), Lille (57.7%), and Liège (55.1%).

Cluster 3 is predominantly composed of students pursuing technology and engineering programs (33.8%; p < .001) who are less likely to have a student job (26.1%; p < .001). These students engage in PA in groups, within enclosed spaces, and at scheduled times (p < .05). The participating institutions with the highest representation in this cluster included Teramo (57.0%), Porto (56.0%), and Rome (54.2%).

Table 4 reports the participating institutions that were significantly associated with cluster membership and therefore contributed to the interpretation of site-level patterns. Given the unequal distribution of respondents across universities, these associations should be interpreted cautiously as exploratory site-level patterns rather than as representative institutional, national, or regional differences.

As illustrated in Table 5, PA in this cluster is performed regularly throughout the year (3.07 ± .96; p < .001) but, typically lasts more than 60 minutes (2.89 ± 1.11; p < .001), and is primarily conducted indoors (2.93 ± 1.21; p < .001).

Pearson’s chi-square tests were conducted to examine associations between cluster membership and categorical variables. Significant associations were found between cluster membership and sex, χ^2^(2, N = 2,165) = 17.54, p < .001, Cramér’s V = .09, indicating a small effect; field of study, χ^2^(12, N = 2,165) = 73.18, p < .001, Cramér’s V = .13, indicating a small effect; student employment, χ^2^(2, N = 2,165) = 50.63, p < .001, Cramér’s V = .15, indicating a small effect; and university, χ^2^(22, N = 2,165) = 1406.82, p < .001, Cramér’s V = .57, indicating a large effect.

The highest proportion of students in the Autonomous cluster was observed in participating institutions located in Prague, Poznań, and Wrocław. The Networked Supervised cluster was more prevalent in Oslo, Dortmund, Lille, and Liège, while the Regularly Supervised cluster was more prevalent in Teramo, Porto, and Rome.

Table 5 shows the over-representation for each quantitative data of each cluster. Table 6 summarizes the main variables that discriminate the three cluster profiles. It should be read together with the detailed statistical results reported in Tables 4 and 5.

**Table 6:** Summary of the discriminative variables, along with considerations for future research.

Cluster	Overall profile	Main discriminative variables	Over-represented characteristics	Considerations for future research
**Cluster 1** Autonomous 42.6%	Flexible, self-organized and less constrained PA participation.	Higher independent practice; more home-based practice; more flexible timing (“at anytime”); more variable frequency throughout the year; more short-duration practice; more practice with an animal; lower supervised practice and lower indoor/structured practice.	Women; humanities/social sciences/arts; students without a job; students from some participating institutions located in Prague, Poznań and Wrocław.	This profile reflects students who tend to engage in PA outside formal or institutional structures. Future studies could examine whether flexible, low-cost, and easily accessible PA opportunities are more acceptable to students displaying this participation pattern.
**Cluster 2** Networked Supervised 28.6%	Supervised and socially/networked practice with outdoor, ICT-supported and longer-duration components.	Higher supervised practice than Cluster 1; higher outdoor activities; higher running/walking, cycling, athletics and water sports; higher ICT communication and functional ICT use; more practice during summer, weekends and holidays; higher durations above 60 or 120 minutes.	Men; sports science students; students with a job; students from some participating institutions located in Oslo, Dortmund, Lille and Liège.	This profile reflects students who combine structured or supervised opportunities with social, digital, and outdoor dimensions. Future studies could evaluate whether adaptable organised activities, digital coordination, and group-based opportunities are associated with uptake or continued participation among students displaying this pattern.
**Cluster 3** Regularly Supervised 28.8%	Regular, structured, indoor and scheduled supervised PA participation.	Highest supervised practice; highest indoor practice; higher regular practice throughout the year; higher practice in autumn, winter and spring; more weekday practice; longer sessions above 60 minutes; lower independent/home/outdoor and short-duration practice.	Technology/engineering students; students less likely to have a job; students from some participating institutions located in Teramo, Rome and Porto.	This profile reflects students whose PA participation is more embedded in regular, structured, and scheduled formats. Future studies could examine whether access to indoor facilities, structured sessions, and predictable schedules is associated with participation among students displaying this pattern.

This table provides an interpretive summary of the main variables that distinguish the three cluster profiles. It should be read alongside the detailed statistical results reported in Tables 4 and 5. The discriminative variables were selected based on significant over- or under-representation within clusters, as well as differences in cluster-specific means or proportions compared to the overall sample. The final column presents hypotheses and considerations for future prospective and intervention research derived from the observed profiles; it should not be interpreted as evidence that the proposed PA formats are effective. This table is intended to facilitate the interpretation of the profiles and does not replace the full statistical information reported in the main results tables.

## Discussion

The present study reveals distinct patterns and differences in how undergraduate students across European universities engage in PA. Two main categories were identified: ‘Autonomous’ and ‘Supervised’. The latter category was further subdivided into ‘Regularly Supervised’ and ‘Networked Supervised’ groups. In this exploratory analysis, participation format and regularity emerged as salient dimensions for distinguishing these profiles, alongside quantity- and intensity-related characteristics.

### The evolution and significance of considering undergraduate students’ preferred formats of experience regarding space, time, and equipment

The literature shows that the intensity, type and amount of PA are important variables for differentiating students (e.g., [21,32]). However, the results of the current study demonstrate that the manner in which PA is engaged, the setting where PA occurs, the time of day and season when PA is performed, and the presence of a pet, the utilization of ICT, or the presence of friends, are significant variables for understanding the involvement of undergraduate students in PA. This finding is noteworthy because it shows that similar levels of PA may involve diverse participation formats. Recent reports and studies have highlighted the prominence of less structured, home-based, and digitally supported forms of PA among young adults [4,30]. Consistent with this literature, the profiles identified in the present study included home-based, pet-related, digitally supported, and free-of-charge practices. Considering the temporal, spatial, social, and equipment-related conditions of participation may therefore complement analyses focused primarily on the quantity of PA.

### Physical activity in new ways

The findings of this study highlight two predominant types of PA practices among European students: supervised activities (predominant in clusters 2 and 3) and self-organized activities (predominant in cluster 1). This pattern aligns with national reports [7] showing that young adults (16–25 years old) increasingly engage in less constrained forms of PA, particularly in terms of space and time [18]. It is also consistent with recent research on emerging forms of self-organized, informal, autonomous, and free PA [33]. Surveys by Croutte and Müller (2021) [18] confirm this trend, indicating that individuals in this age group are progressively favoring less structured formats that offer greater autonomy over space, time, and scheduling.

Our results further show no statistically significant differences in the types of activities practised across demographic clusters. Previous studies [7,18] have documented a growing diversification of activities among undergraduate students, with many trying new forms of PA or engaging in multiple sports simultaneously, regardless of material constraints. This shift is especially pronounced among those moving away from conventional or institutionalised sports.

One potential explanation for these changing participation patterns is the institutionalisation of PA, which may: (1) offer less flexibility than home-based activities [4]; (2) require more time, 41% of 16–25-year-olds report insufficient time to engage in sport [4]; and (3) entail higher costs, 10% consider sport too expensive [4]. In contrast, unstructured PA offers autonomy, adaptability to personal schedules, and alignment with individual motivations such as maintaining physical and mental health [34]. Previous research suggests that flexible PA formats may be associated with more personalised or enjoyable experiences and with continued participation [35]; however, these outcomes were not evaluated in the present study. Structured activities provide opportunities for social interaction and organised routines, whereas unstructured formats may be more compatible with independence and individual scheduling preferences. The coexistence of supervised and unsupervised participation profiles suggests a need to examine whether PA opportunities adapted to different preferences influence uptake, enjoyment, or continued participation.

From students’ perspectives, financial constraints remain a significant barrier to PA participation [36]. Nevertheless, conventional supervised activities remain highly popular, with 57.4% of surveyed students over-represented in this category. These factors shape individual PA habits [37] and are particularly relevant for the 35% of Europeans who have never participated in PA [7]. Given that the transition to higher education represents a critical life stage for PA participation [8,10], the varied participation profiles identified in this study suggest that inclusive programmes accommodating different forms of PA warrant consideration and prospective evaluation.

### Physical activity and differences across participating institutions

Our findings suggest that PA practices varied across the participating institutions. Cluster membership was therefore significantly associated with the participating institution. However, given the uneven distribution of respondents across universities, these associations should be interpreted with caution as exploratory, site-level patterns rather than as representative of institutional, national or regional differences. Students from the participating universities located in Prague, Poznań, and Wrocław were more represented in the Autonomous cluster (Cluster 1), whereas students from some other participating institutions were more represented in the supervised clusters. These observations align with Frömel et al. (2022) [38], who reported that Czech and Polish boys preferred individual activities, often performed in natural settings or water-based environments.

Cluster 2 (‘Networked Supervised’) was observed across several participating institutions. Finally, Cluster 3 (“Regularly Supervised”) had a higher representation of students from participating universities in Teramo, Rome, and Porto, indicating a greater involvement in organized activities with peers during the academic year.

Furthermore, Gerovasili et al. (2015) [20] showed that time spent on sporting activities declines from northern to western and southern Europe. Consistently, Lera-Lopez and Marco (2021) [21] identified significant regional disparities in PA levels across 196 European regions, emphasizing the role of socioeconomic and cultural factors. They suggest that European regional coordination; through collaborative use of sports infrastructure, co-financing inter-regional facilities, shared physical education programs, and inter-regional sporting events; could reduce disparities and enhance health-promoting PA. While these regional effects should be interpreted with caution, these studies provide useful contextual background for interpreting differences between participating institutions. However, the current study was not designed to test cultural or regional mechanisms directly, and the observed patterns should therefore be interpreted as exploratory associations rather than causal evidence of broader regional differences. Future research using balanced samples across institutions and countries, together with multilevel designs, is needed to examine whether the observed profiles remain stable across different institutional and geographic contexts.

### Physical activity mode and sex

Our results reveal a distinction between cluster 1 (‘Autonomous’, with a significantly higher representation of women) and cluster 2 (‘Networked Supervised’, with a significantly higher representation of men). This suggests that there are significant differences (p < .05) in PA preferences between sexes, with women exhibiting a tendency (over-represented in cluster 1: 51.3%) to engage in independent PA, potentially alongside pets or friends. However, this association may partly reflect differences in sample composition across participating institutions and should not be generalised to the broader European undergraduate population. At the European level, recent studies have shown that girls tend to favour less intense PA compared to boys [38]. Additionally, research by Croutte and Müller (2021) [18] has demonstrated that men tend to dominate collective exercise spaces. However, the present study did not directly assess perceived comfort, safety, belonging, or barriers related to structured or indoor PA settings. Therefore, the overrepresentation of women in the ‘Autonomous’ cluster (cluster 1) should not be interpreted as evidence of discomfort in structured or indoor PA settings. Instead, this pattern raises the question of whether flexible, self-organised, or less constrained PA formats are perceived differently by women and men, which should be examined directly in future research.

Future research could examine whether students perceive PA sessions organised in smaller groups, outdoor settings, or other formats differently, and whether these formats are associated with enjoyment, uptake, or continued participation. As individual preferences vary widely [30], the effects of such organisational arrangements should be evaluated rather than assumed. Prospective and intervention studies are also needed to test whether the experiential and pedagogical characteristics of structured activities influence these outcomes [10]. Given the small association between sex and cluster membership, it would be unwise to assume that any particular format is preferable or effective for women or men.

### Limitations

A primary limitation of the current study is the significant variation in the number of respondents from different universities, as well as the use of a convenience-based recruitment strategy. Since some institutions contributed a greater number of responses than others, the resulting clusters and associations may reflect site-specific recruitment dynamics or institutional contexts. Therefore, the sample should be considered a convenience sample rather than a representative sample of European undergraduate students. The findings should be interpreted as exploratory, site-level patterns rather than as representative estimates of broader institutional, national, or regional differences. Additionally, participants were nested within universities and countries; however, this multilevel structure was not formally modeled in the exploratory clustering approach. Consequently, the associations between cluster membership and variables such as sex, field of study, employment status or university should be interpreted with caution, as they may partly reflect site-specific recruitment dynamics or institutional contexts. Future studies should use more balanced sampling strategies and consider multilevel or robust inference approaches when examining whether these profiles remain stable across universities and countries. In addition, important variables were not considered in this study, such as regional economic factors, tertiary education rates, social use of the internet [21], and access to sports infrastructure [39]. Additionally, the academic programs followed by the students and their perceived difficulty were not covered. A more comprehensive examination of these dimensions could provide a better understanding of how individual, institutional, and environmental constraints are associated with undergraduate students’ PA participation, in line with Newell’s (1986) [40] model of constraints or systemic approaches.

### Implications for future research and practice

The findings raise several considerations for addressing the increasingly sedentary lifestyles of undergraduate students. Given the diversity of participation profiles identified, it may be worthwhile to consider offering a range of physical activity (PA) opportunities adapted to different needs and to conduct a prospective evaluation. Notably, 42.6% of participants belonged to the Autonomous cluster. Therefore, future research could examine the barriers to and facilitators of self-organized PA. Potential avenues include campaigns, events, and attention to the physical and structural environments of universities and their surrounding areas, particularly in areas where students live and study. These possibilities are consistent with the ecological framework [12], which emphasizes the interaction between individuals and their environments. However, the present study did not assess the effects of these interactions on PA participation. Another potential approach to examine is the systematic integration of compulsory sports credits into university curricula, particularly in relation to autonomous practice and individualized PA opportunities.

In countries such as Germany, France, and Denmark, universities provide access to a broad range of affordable sports courses through dedicated sports programs. These existing models illustrate different ways of offering PA opportunities that may correspond to varied student preferences. Future prospective and intervention studies should evaluate whether such provisions facilitate the integration of PA into students’ daily routines and support continued participation over time.

## Conclusion

This study illuminates the varied landscape of PA engagement among undergraduate students at participating European universities. By identifying three distinct clusters: ‘Autonomous,’ ‘Networked Supervised,’ and ‘Regularly Supervised’, the findings underscore the importance of considering not only the quantity and intensity of PA, but also how, where, when, and under what social and organisational conditions PA is practised.

Our results contribute to the existing body of knowledge by illustrating the dynamic nature of PA preferences among undergraduates. Tailored approaches, such as offering diverse activity options and reimagining PA and sports pathways, may warrant consideration and evaluation in future research as ways of responding to students’ varied behaviours and preferences. Universities could examine whether creating more inclusive and flexible opportunities that align with students’ lifestyles is associated with greater engagement or healthier routines.

Additionally, the differences observed across participating institutions suggest that the relevance of flexible and context-sensitive approaches should be examined prospectively. These exploratory findings do not support a single approach to promoting PA participation. Instead, a nuanced understanding of students’ preferences and needs may help formulate hypotheses for prospective and intervention studies conducted during the transition from secondary school to higher education.

The findings also reveal sex differences in PA engagement. These results do not support recommendations for a specific sex-targeted PA format. Future research could examine whether organisational arrangements such as smaller group sizes, outdoor settings, or other activity formats are perceived differently by women and men and whether they are associated with participation, comfort, or belonging.

Furthermore, the study underscores the importance of considering how PA is experienced and the conditions under which participation occurs when examining undergraduate students’ preferences. The acceptability and potential effects of offering varied exercise formats and opportunities for autonomous participation should be evaluated in future prospective and intervention studies. These insights may help generate hypotheses and inform the design and evaluation of future university initiatives and public policies aimed at reducing sedentary lifestyles.

Finally, investigating PA based on the types of experiences and practices undergraduate students seek provides a broader framework for understanding participation beyond quantity and intensity alone. Whether offering PA in varied formats increases students’ PA levels, supports continued participation, or contributes to healthier routines should be tested through prospective and intervention research.

## Supporting information

S1 File Internal validation indices for alternative cluster solutions. ^a^ Number of clusters tested in alternative clustering solutions. ^b^ Average silhouette width measures the cohesion and separation of clusters, with higher values indicating better-defined clusters. ^c^ The gap statistic compares the observed clustering structure with a reference distribution; higher values indicate a stronger clustering structure relative to random data. ᵈ The standard error of the gap statistic (Gap SE). ^e^ The final decision was based on internal validation indices, parsimony, cluster size and substantive interpretability. Although no single statistical optimum was identified, the three-cluster solution was retained as the best substantive compromise.
(DOCX)

S2 File Explanatory notes for tables. Where, when, how, and with whom do young university students engage in physical activity?
(DOCX)
