# Supplementary material for: Where, when, how, and with whom do young university students engage in physical activity? A cluster-derived spectrum of patterns across 16 European universities
Source: PLoS One. 2026 Nov 4;21(11):e0360751. doi: 10.1371/journal.pone.0360751 (PMC13734477; doi:10.1371/journal.pone.0360751)
Supplement: Supplementary file 1 [file pone.0360751.s001.docx]

**Supplementary Information 1. Internal validation indices for alternative cluster solutions.**

| **Number of clusters** ^a^ | **Average silhouette width** ^b^ | **Gap statistic** ᶜ | **Gap SE** ᵈ | **Decision** ^e^ |
| --- | --- | --- | --- | --- |
| 2 | 0.201 | 1.285 | 0.006 | Alternative solution not retained |
| **3** | **0.191** | **1.337** | **0.006** | **Retained: best substantive compromise** |
| 4 | 0.160 | 1.355 | 0.006 | Alternative solution not retained |
| 5 | 0.140 | 1.371 | 0.006 | Alternative solution not retained |
| 6 | 0.131 | 1.377 | 0.007 | Alternative solution not retained |

^a^ Number of clusters tested in alternative clustering solutions.

^b^ Average silhouette width measures the cohesion and separation of clusters, with higher values indicating better-defined clusters.

ᶜ The gap statistic compares the observed clustering structure with a reference distribution; higher values indicate a stronger clustering structure relative to random data.

ᵈ The standard error of the gap statistic (Gap SE).

^e^ The final decision was based on internal validation indices, parsimony, cluster size and substantive interpretability. Although no single statistical optimum was identified, the three-cluster solution was retained as the best substantive compromise.
