# Supplementary material for: Where, when, how, and with whom do young university students engage in physical activity? A cluster-derived spectrum of patterns across 16 European universities
Source: PLoS One. 2026 Nov 4;21(11):e0360751. doi: 10.1371/journal.pone.0360751 (PMC13734477; doi:10.1371/journal.pone.0360751)
Supplement: Supplementary file 2 [file pone.0360751.s002.docx]

**Supplementary Information 2. Explanatory notes for tables: Where, when, how, and with whom do young university students engage in physical activity?**

# Purpose of this supplementary file

This supplementary file was prepared to improve transparency and interpretability of the tables reported in the manuscript. It explains the purpose of each table, how the values should be read, and how each table contributes to the interpretation of the cluster-derived profiles.

# Overview of supplementary tables

| **Supplementary item** | **Content** | **Main purpose** | **How it supports interpretation** |
| --- | --- | --- | --- |
| S1  **Table 1. Characteristics of the participants.** | Characteristics of the participants | Describes the overall sample. | Provides the socio-demographic context of the analytical sample. |
| S2  **Table 2. Description of respondents included in the study, by country.** | Respondents by university/country | Documents the recruitment distribution across sites. | Shows the sampling imbalance and helps contextualize country or university patterns. |
| S3  **Table 3. Significant correlations between variables and dimensions identified through factorial analysis of mixed data (FAMD).** | Factor loadings/correlations on the dimensions | Identifies the variables structuring the factorial space. | Clarifies the dimensions used to interpret the profiles. |
| S4  **Table 4.** **Characteristics for each cluster of qualitative data (in %)** | Qualitative cluster characteristics | Compares categorical variables across clusters. | Shows over- or under-representation of sex, field of study, employment, and university. |
| S5  **Table 5. Characteristics for each cluster of quantitative data (ordinal scale from 1 to 5).** | Quantitative cluster characteristics | Compares ordinal/quantitative variables across clusters. | Shows the practice formats, timing, context, ICT use, and duration that distinguish clusters. |
| S6  **Table 6. Summary of the discriminative variables, along with a practical interpretation of the clusters.** | Summary of the main variables | A synthetic overview of the most discriminative variables for each cluster. | Helps to interpret the three cluster-derived profiles by linking the detailed statistical results from Tables 4 and 5 to a clear practical interpretation. |

**Detailed notes**

## S1 – Table 1. Characteristics of the participants

**Purpose:** This table describes the final analytical sample included in the study. It reports the distribution of participants by sex, field of study, employment status, and age.

**How to read:** Categorical variables are presented as n (%). Age is presented as mean ± standard deviation.

**Interpretation:** This table should be used to understand the composition of the sample before interpreting cluster membership. It also shows that women were more represented than men in the final sample, which should be considered when interpreting sex-related cluster differences.

| **Variable** | **Ecological level / dimension** | **Type** | **Definition** | **Response or coding information** | **Role in analysis** |
| --- | --- | --- | --- | --- | --- |
| Sex | Individual | Categorical | Participant-reported sex category used to describe cluster membership. | Man or woman | Used as an illustrative/profiling variable for cluster interpretation. |
| Age | Individual | Continuous | Participant age at questionnaire completion. | Years; eligibility restricted to the study age range. | Used to describe the sample and eligibility. |
| Field of study | Individual/academic context | Categorical | Participant academic field or program category. | Categories include law/political science, business/management, medicine, sports science, humanities/social sciences/arts, technology/engineering, other field. | Used to describe the sample and characterize cluster membership. |
| Employment status | Individual/contextual | Categorical | Whether the participant reported having a student job/employment. | Yes/No or equivalent final coding. | Used to describe the sample and characterize cluster membership. |

## S2 – Table 2. Description of respondents included in the study, by university/country

**Purpose:** This table reports the number and percentage of respondents from each participating university or country grouping.

**How to read:** Values indicate the contribution of each site to the final analytical sample.

**Interpretation:** This table is essential for transparency because respondents were not evenly distributed across universities. Some sites contributed substantially more participants than others. Therefore, university- or country-level patterns should be interpreted cautiously as exploratory associations rather than representative estimates of European undergraduate students.

| **Variable** | **Ecological level / dimension** | **Type** | **Definition** | **Response or coding information** | **Role in analysis** |
| --- | --- | --- | --- | --- | --- |
| University/country | Institutional context | Categorical | Participating university or institutional site. | **Name of university from the list :** University of Teramo (Italy), University College Cork (Ireland), University of Prague (Czech Republic), Norwegian School of Sport Sciences, Oslo (Norway), University of Stirling (Scotland), Catholic University of Leuven (Belgium), Ghent University (Belgium), University of Geneva (Switzerland), HEP Vaud (Switzerland), Technical University of Dortmund (Germany), University of Liège (Belgium), University of Southern Denmark (Denmark), Sapienza University of Rome (Italy), Adam Mickiewicz University, Poznań (Poland), University of Kent (England), University of Huelva (Spain), University School of Physical Education, Wrocław (Poland), University of Lille (France), University of Porto (portugal), Other. | Used as a contextual marker and to examine cluster. |

S3 – **Table 3. Significant correlations between variables and dimensions identified through factorial analysis of mixed data (FAMD).**

**Purpose:** This table identifies the variables most strongly associated with the first two dimensions of the mixed-data factorial analysis.

**How to read:** Positive and negative values indicate the direction and strength of association between variables and dimensions. Variables with stronger absolute values contribute more to the interpretation of the dimension.

**Interpretation:** Dimension 1 mainly contrasts structured, supervised, scheduled, and year-round practice with free, home-based, shorter, and more variable forms of practice. Dimension 2 mainly reflects outdoor, autonomous, ICT-supported, weekend/holiday-oriented practice, contrasted with indoor/private/registered or more constrained practice contexts.

## S4 – Table 4. Characteristics for each cluster of qualitative data (in %).

**Purpose:** This table compares categorical characteristics across the three clusters, including sex, field of study, employment status, and university.

**How to read:** Percentages indicate the proportion of a given category represented in each cluster.

**Interpretation:** This table helps identify which categories are over- or under-represented in each cluster.

## S5 – Table 5. Characteristics for each cluster of quantitative data (ordinal scale from 1 to 5).

**Purpose:** This table compares the mean scores of ordinal/quantitative variables across the three clusters. It describes practice formats, periods of practice, types of activities, ICT use, and exercise duration.

**How to read:** Results are presented as mean ± standard deviation. Higher values indicate stronger endorsement or higher reported involvement in the corresponding practice format.

**Interpretation:** This table provides the empirical basis for naming and interpreting the clusters.

- Cluster 1 is mainly characterized by autonomous, flexible, home-based, and less constrained practices.
- Cluster 2 combines supervised/networked practice with outdoor activities, ICT use, and longer or more varied participation.
- Cluster 3 is characterized by more regular, structured, indoor, and scheduled supervised practice.

These variables correspond to the quantitative indicators reported in Table 5 of the manuscript:

| **Variable label in manuscript** | **Analytical dimension** | **Type** | **Definition / what the variable captures** | **Response or coding information** | **Interpretive role** |
| --- | --- | --- | --- | --- | --- |
| Independent practice | Organisation / social context | Ordinal | Preference or involvement in PA performed independently, without direct supervision. | Higher values indicate stronger endorsement or higher reported involvement. | Discriminates autonomous and less supervised forms of PA. |
| Supervised practice | Organisation / social context | Ordinal | Preference or involvement in PA performed under supervision, coaching, instruction, or formal guidance. | Higher values indicate stronger endorsement or higher reported involvement. | Key marker of structured/supervised clusters. |
| Practice indoors | Spatial context | Ordinal | Preference or involvement in PA performed in indoor facilities or enclosed spaces. | Higher values indicate stronger endorsement or higher reported involvement. | Distinguishes structured indoor profiles from autonomous/home/outdoor profiles. |
| Practice at home | Spatial context | Ordinal | Preference or involvement in PA performed at home or in the domestic environment. | Higher values indicate stronger endorsement or higher reported involvement. | Key marker of autonomous and flexible PA participation. |
| Outdoor activities | Spatial context | Ordinal | Preference or involvement in PA performed outdoors. | Higher values indicate stronger endorsement or higher reported involvement. | Helps distinguish less constrained or environment-based PA formats. |
| With an animal | Social/contextual arrangement | Ordinal | Preference or involvement in PA performed with an animal, such as walking a dog. | Higher values indicate stronger endorsement or higher reported involvement. | Associated with autonomous and everyday-life PA formats. |
| In autumn | Seasonality | Ordinal | Preference or involvement in PA during autumn. | Higher values indicate stronger endorsement or higher reported involvement. | Seasonal timing variable contributing to cluster profiles. |
| In summer | Seasonality | Ordinal | Preference or involvement in PA during summer. | Higher values indicate stronger endorsement or higher reported involvement. | Seasonal timing variable contributing to cluster profiles. |
| In winter | Seasonality | Ordinal | Preference or involvement in PA during winter. | Higher values indicate stronger endorsement or higher reported involvement. | Seasonal timing variable contributing to cluster profiles. |
| In spring | Seasonality | Ordinal | Preference or involvement in PA during spring. | Higher values indicate stronger endorsement or higher reported involvement. | Seasonal timing variable contributing to cluster profiles. |
| During the week | Temporal context | Ordinal | Preference or involvement in PA during weekdays (Monday to Friday). | Higher values indicate stronger endorsement or higher reported involvement. | Distinguishes regular/scheduled participation from less constrained formats. |
| On weekends | Temporal context | Ordinal | Preference or involvement in PA during weekends (Saturday and Sunday). | Higher values indicate stronger endorsement or higher reported involvement. | Identifies leisure-time or non-academic-time PA formats. |
| During holidays | Temporal context | Ordinal | Preference or involvement in PA during holidays or vacation periods. | Higher values indicate stronger endorsement or higher reported involvement. | Highlights PA formats less tied to academic routines. |
| At anytime | Temporal flexibility | Ordinal | Preference or involvement in PA without fixed or constrained timing. | Higher values indicate stronger endorsement or higher reported involvement. | Key marker of autonomous/flexible participation. |
| Regular throughout the year | Regularity | Ordinal | Preference or involvement in PA maintained regularly across the year. | Higher values indicate stronger endorsement or higher reported involvement. | Key marker of regular and structured profiles. |
| Variable throughout the year | Regularity | Ordinal | Preference or involvement in PA with variable frequency across the year. | Higher values indicate stronger endorsement or higher reported involvement. | Associated with flexible or less institutionalised PA patterns. |
| Running/walking | Activity type | Ordinal | Preference or involvement in running and/or walking activities. | Higher values indicate stronger endorsement or higher reported involvement. | Activity-type variable contributing to cluster interpretation. |
| Cycling | Activity type | Ordinal | Preference or involvement in cycling. | Higher values indicate stronger endorsement or higher reported involvement. | Activity-type variable contributing to cluster interpretation. |
| Athletics | Activity type | Ordinal | Preference or involvement in athletics-related activities (Track and field). | Higher values indicate stronger endorsement or higher reported involvement. | Activity-type variable contributing to cluster interpretation. |
| Water sports | Activity type | Ordinal | Preference or involvement in water-based activities. | Higher values indicate stronger endorsement or higher reported involvement. | Activity-type variable contributing to cluster interpretation. |
| ICT Communication | Equipment / digital tools | Ordinal | Use of digital tools to exchange information, coordinate practice, or share PA-related experiences with others. | Higher values indicate greater use of communication-related ICT. | Digital/social coordination marker. |
| Functional ICT | Equipment / digital tools | Ordinal | Use of digital tools to guide, monitor, plan, or organize PA, such as online programs, applications, or tracking devices. | Higher values indicate greater use of functional ICT. | Digital self-management and monitoring marker. |
| Less than 30 min | Duration | Ordinal | Preference or involvement in PA sessions lasting less than 30 minutes. | Higher values indicate stronger endorsement or higher reported involvement. | Short-duration participation marker. |
| Less than 60 min | Duration | Ordinal | Preference or involvement in PA sessions lasting less than 60 minutes. | Higher values indicate stronger endorsement or higher reported involvement. | Short/moderate-duration participation marker. |
| More than 60 min | Duration | Ordinal | Preference or involvement in PA sessions lasting more than 60 minutes. | Higher values indicate stronger endorsement or higher reported involvement. | Longer-duration participation marker. |
| More than 120 min | Duration | Ordinal | Preference or involvement in PA sessions lasting more than 120 minutes. | Higher values indicate stronger endorsement or higher reported involvement. | Very long-duration participation marker. |
