# Supplementary material for: Where, when, how, and with whom do young university students engage in physical activity? A cluster-derived spectrum of patterns across 16 European universities
Source: PLoS One. 2026 Nov 4;21(11):e0360751. doi: 10.1371/journal.pone.0360751 (PMC13734477; doi:10.1371/journal.pone.0360751)
Supplement: Supplementary file 3 [file pone.0360751.s004.docx]

Title of manuscript: **Where, when, how, and with whom do young university students engage in physical activity? A cluster-derived spectrum of patterns across 16 European universities.**

Dear Professor Emily Chenette,

We would like to thank you and the reviewers sincerely for carefully evaluating our manuscript and providing constructive and insightful comments. In response to the major points raised, we have thoroughly revised the manuscript and addressed each comment in detail.

In particular, we have substantially strengthened the methodological and statistical reporting. We clarified the analytical pipeline, including the use of factor analysis of mixed data, hierarchical clustering, and k-means consolidation. We revised the reporting of missing data, outlier handling, cluster validation, and statistical tests. We now report chi-square statistics, degrees of freedom, sample size, p-values, and Cramér’s V effect sizes more clearly.

Additionally, we carefully revised the interpretation of country, regional, and university-level patterns. To avoid overinterpretation, the manuscript no longer presents these findings as evidence of broad cultural or regional differences. Instead, they are now described as exploratory, institutional-level associations. Additional caution has been added in the Discussion and Limitations sections regarding the imbalance in recruitment and the nested structure of the data.

Several additional revisions were made to improve transparency and reproducibility. We updated the data availability statement, clarified the ethical reporting, and added supplementary materials, including variable definitions, coding information, explanatory notes for tables, a concise summary of discriminative variables by cluster, and internal validation indices for the cluster solution.

We revised the manuscript for English grammar, clarity, and consistency of terminology. All changes made in response to the reviewers' concerns are highlighted in the revised manuscript: blue for Reviewer 1, green for Reviewer 2, and orange for both.

We hope that these revisions adequately address the reviewers’ comments and improve the clarity, rigor, and reproducibility of the manuscript. We sincerely appreciate the opportunity to revise and resubmit our manuscript to PLOS ONE.

Yours sincerely,

The Authors

Date of resubmission: 8 June 2026

**Revisions in Response to Comments from Reviewer 1:**

| ***Comment/feedback from peer review:***  In this column, please state the reviewer’s feedback verbatim. | ***Author(s) change:***  In this column, please include your change verbatim. | ***Location:***  In this column, please provide the line number(s) and page of the author(s) change. | ***Additional author(s) notes about the change*** |
| --- | --- | --- | --- |
| Thank you for the opportunity to review this manuscript, which examines where, when, how, and with whom first-year university students engage in physical activity across 16 European universities. The cross-national scope and intention to characterize patterns beyond simple participation levels are valuable. |  |  | We would like to thank the reviewer for their careful and constructive evaluation of our manuscript. We are pleased that the reviewer recognised the value of the cross-national scope and our attempt to characterise patterns of participation in PA beyond simple participation levels. We have revised the manuscript carefully to improve methodological transparency and reproducibility, as well as ethical and statistical reporting and the cautious interpretation of the findings. |
| **1.** A central requirement that must be strengthened is the availability and reproducibility of data. The current information on data availability does not clearly specify a concrete public location for the dataset underlying the analyses, such as a repository link, a DOI, or an accession number. To meet PLOS ONE requirements, the authors should deposit the complete de-identified dataset and an accompanying data dictionary or codebook in an appropriate public repository, provide a stable URL, DOI, or accession number in the Data Availability Statement, and share the analysis code needed to reproduce the dimensionality reduction and clustering workflow, as well as the reported tables and figures. If there are legitimate restrictions on sharing data due to privacy or third-party conditions, these restrictions should be explicitly stated, and the minimum necessary shareable dataset and documentation should still be provided. |  |  | We thank the reviewer for this important comment. We have revised the Data Availability Statement to clarify data access and reproducibility. The complete de-identified dataset, along with the relevant data dictionary/codebook has been deposited on the Recherche Data Gouv repository (DOI: 10.57745/HKG3H8).  In accordance with repository and institutional procedures, access to the dataset is currently restricted to preserve author anonymity while the article is under review. You may request access through the repository by contacting the study investigators. The dataset will be made publicly available upon publication. |
| **2.** The manuscript also requires clearer and more consistent ethical reporting. The ethics information appears inconsistent regarding the name of the approving ethics committee. The authors should ensure that the approving body is correctly and consistently identified throughout the manuscript and the supporting documents. In addition, it should be clarified whether a single ethics approval covered the multi-country data collection or whether additional approvals and authorizations were required across partner institutions. The consent procedure should be described consistently and in sufficient detail to allow readers to understand how informed consent was obtained and managed at each site. | The following sentence: “Prior to completing the questionnaire, participants provided informed consent and were instructed to do so in a quiet environment. Ethical approval was granted by the Behavioural Sciences Ethics Committee of X (approval number 2021-475-S91)” has been replaced by the following paragraph: “The study protocol was reviewed and approved by the Ethics Committee for Experimental Human Sciences at the University of Lille in France (CER-Lille; approval number 2021-475-S91) corresponding to the HYPEM project. It was conducted as a multi-site European study, coordinated by the University of Lille. This central ethics approval covered the overall research protocol, including the online questionnaire, consent procedure and data management plan. As recruitment and dissemination were conducted through partner universities, each institution was responsible for ensuring compliance with its own local institutional procedures and applicable national or regional regulations before distributing the questionnaire to students.  Prior to completing the questionnaire, participants were provided with information regarding the study's objectives, the voluntary nature of their participation, the data protection procedures in place, and their rights as participants. Electronic informed consent was obtained prior to questionnaire completion.” (Line 183)  **This revision has been made in the Methods section.** | Page 10,  Materials and methods,  Data collection procedure,  Lines 183 to 195. | The study protocol was reviewed and approved by the Ethics Committee for Experimental Human Sciences at the University of Lille in France (CER-Lille; approval number 2021-475-S91; favourable decision issued on 9 March 2021).  The study is linked to the HYPEM project which was coordinated as a European multi-site study, with the University of Lille acting as the coordinating institution. Central ethics approval covered the overall study protocol, including the online questionnaire, data collection procedure, consent process and data management plan. As recruitment and dissemination were conducted through partner universities in different countries, each participating institution was responsible for complying with its own local institutional procedures and applicable national or regional regulations before disseminating the questionnaire to students.  The consent procedure has also been clarified in the manuscript. Before accessing the questionnaire, participants were provided with information about the study objectives, voluntary participation, data protection and their rights as participants. They provided electronic informed consent before completing the questionnaire. |
| **3.** The Methods section would benefit from clearer terminology and a more transparent description of the analytical pipeline for mixed data dimensionality reduction and clustering. Currently, terms such as exploratory factor analysis of mixed data are presented alongside a workflow that includes PCA-based steps, hierarchical clustering, and K-means clustering, which can be confusing without precise specifications. The authors should clearly state which method was used to handle mixed data, for example, FAMD, MCA, or a specific PCA variant, and explain how categorical and ordinal variables were encoded, scaled, and weighted. | We changed all the **‘Methods – analysis’** section: “Prior to conducting the multivariate analyses, the dataset was screened for missing values, inconsistent responses and outliers. The extent and pattern of missing data were first examined. Participants with incomplete questionnaires or responses that did not meet the inclusion criteria were excluded from the multivariate analyses using a complete-case procedure. This resulted in a final analytic sample of N = 2,165 participants. Extreme values were examined at the univariate level for quantitative variables only, with cases being excluded when values were considered implausible or inconsistent with the study criteria. This screening process resulted in the exclusion of 124 observations. No multivariate outlier detection procedure was applied to the mixed dataset. To assess the robustness of the exclusion procedure, the clustering analysis was repeated with and without the excluded observations. The resulting cluster structure, composition and interpretation remained highly consistent.  To handle the mixed structure of the dataset, Factor Analysis of Mixed Data (FAMD) was conducted using the FactoMineR package in R (Husson et al., 2016), which allows continuous, categorical and ordinal variables to be analysed within a single analytical framework. This analytical strategy aligns with previous applications of FAMD and cluster analysis in studies examining the PA participation profiles of female university students (Fermon et al., 2026). Continuous variables were standardised and categorical variables were encoded using a complete disjunctive indicator coding scheme. Ordinal variables were treated according to their ordered response scales. The FAMD procedure automatically balances the contribution of different variable types, thereby reducing the risk of one type of variable dominating the analysis. The resulting principal dimensions were then used as input for the clustering procedure.  First, hierarchical agglomerative clustering was performed to identify natural groupings in the data. The number of clusters was determined by examining the hierarchical clustering dendrogram, changes in within-cluster inertia, cluster size and the interpretability of alternative solutions. Based on these criteria, a three-cluster solution was retained. To further assess the robustness of the final solution, internal validation indices were computed for alternative solutions with two to six clusters. The average silhouette width and gap statistics were examined alongside cluster size and substantive interpretability. While these indices did not identify a single statistical optimum, the three-cluster solution offered the best balance between internal validation indices, parsimony, cluster size and interpretability. These validation indices are reported in Supplementary Table A. The final solution was then consolidated using the K-means algorithm, which refines cluster membership by minimising within-cluster variance.  Cluster interpretation was based on comparisons between cluster-specific means or proportions of categorical variables and the corresponding values in the overall sample. The V-test statistic was used to identify variables that were significantly over- or under-represented in each cluster based on their deviation from the overall sample distribution (Lebart et al., 2006). Where appropriate, descriptive statistics were reported, including cluster-specific means, proportions, standard deviations, v-test values and significance levels. Statistical significance was set at p < .05.  The adequacy of the sample for factor analysis was confirmed using the Kaiser–Meyer–Olkin (KMO) index, which indicated good suitability (KMO = 0.849). All analyses were conducted using R Studio (version 4.3.0) with the following relevant packages: rstatix, FactoMineR, FactoShiny, FactoInvestigate and Psych.” | Page 11 and 12,  Materials and methods,  Analysis,  Lines 197 to 238. | We thank the reviewer for this valuable feedback, which has helped us to substantially improve the clarity and transparency of the statistical procedures.  We have revised all the 'Methods' section to provide a clearer and more detailed description of the data pre-processing stage and the full analytical pipeline.  Firstly, we have clarified how missing data and inconsistent responses were handled. Participants with incomplete questionnaires or responses that did not meet the inclusion criteria were excluded prior to the multivariate analyses, using a complete-case approach. This resulted in a final analytical sample of N = 2,165 participants.  Secondly, we revised the description of outlier handling. In response to the reviewer’s concern, we have removed the reference to Grubbs' test and clarified that extreme values were screened at the univariate level for quantitative variables only. Cases were excluded only when values were considered implausible or inconsistent with the study criteria. This screening process resulted in the exclusion of 124 observations. We also conducted a sensitivity analysis by repeating the clustering procedure with and without these excluded cases. The resulting cluster structure, composition and interpretation remained highly consistent, which supports the robustness of the findings.  Finally, we have clarified the analytical workflow for mixed data. The revised manuscript explicitly states that Factor Analysis of Mixed Data (FAMD), implemented in the FactoMineR package in R, was used to handle the mixed structure of the dataset. We explain how we handled continuous, categorical and ordinal variables, and clarify that we subsequently used the FAMD dimensions for hierarchical agglomerative clustering, followed by K-means consolidation. We hope that these changes address the reviewer’s concerns regarding methodological clarity, transparency, and reproducibility. |
| **4.** The handling of missing data needs to be explicitly documented, including the amount of missingness, assumptions about the missingness mechanism, and whether imputation or listwise deletion was used to address missing data.  **5.** The approach to outlier handling also requires stronger justification, including how many observations were excluded, why this decision was made, and whether key results remained stable when the analysis was repeated with and without these exclusions. | We expanded the **‘Methods’** (analysis section) section to provide further details on the outlier-screening procedure: “Prior to conducting the multivariate analyses, the dataset was screened for missing values, inconsistent responses and outliers. The extent and pattern of missing data were first examined. Participants with incomplete questionnaires or responses that did not meet the inclusion criteria were excluded from the multivariate analyses using a complete-case procedure. This resulted in a final analytic sample of N = 2,165 participants.” (Line 197)  Extreme values were examined at the univariate level for quantitative variables only, with cases being excluded when values were considered implausible or inconsistent with the study criteria. This screening process resulted in the exclusion of 124 observations. No multivariate outlier detection procedure was applied to the mixed dataset. To assess the robustness of the exclusion procedure, the clustering analysis was repeated with and without the excluded observations. The resulting cluster structure, composition and interpretation remained highly consistent.” (Line 197)  We also revised the **'Participants'** section to provide the following clarification: “Of the 4,321 undergraduate students who responded to the survey, 2,165 participants (1,413 females) were retained in the final analytic sample after applying the inclusion criteria, complete-case selection, and data-screening procedures.” (Line 135) | Page 11 and 12  Materials and methods,  Analysis,  Lines 197 to 207.  Page 7,  Materials and Methods, Participants,  Line 135 to 137. | We thank the reviewer for pointing out this omission. We have added a statement dedicated to missing data handling.  We have also revised the Methods section to clarify that outlier screening was conducted prior to the multivariate analyses and was limited to extreme values at the univariate level.  → Same response for comment 8 to reviewer 2. |
| **6.** The rationale for selecting the number of clusters should be strengthened. A scree plot or Kaiser criterion is not typically used as a primary justification for the number of clusters, and cluster number selection should be supported by appropriate validation.  The authors should include internal validation metrics, such as silhouette or gap statistics, and evaluate cluster stability using bootstrapping or split-sample validation. The manuscript should report whether the three-cluster solution remains robust under reasonable alternative analytical choices, including different seeds, distance measures, and clustering algorithms. | We added in the **‘Methods’** (analysis section) section: “To handle the mixed structure of the dataset, Factor Analysis of Mixed Data (FAMD) was conducted using the FactoMineR package in R (Husson et al., 2016), which allows continuous, categorical and ordinal variables to be analysed within a single analytical framework. This analytical strategy aligns with previous applications of FAMD and cluster analysis in studies examining the PA participation profiles of female university students (Fermon et al., 2026).” (Line 208)  We added in the **‘Methods’** (analysis section) section: “To further assess the robustness of the final solution, internal validation indices were computed for alternative solutions with two to six clusters. The average silhouette width and gap statistics were examined alongside cluster size and substantive interpretability. While these indices did not identify a single statistical optimum, the three-cluster solution offered the best balance between internal validation indices, parsimony, cluster size and interpretability. These validation indices are reported in Supplementary Table A.” (Line 221). | Page 11,  Materials and methods,  Analysis,  Lines 208 to 212.  Page 11,  Materials and methods,  Analysis,  Lines 221 to 227. | This point was also raised by the other reviewer, who noted that the Kaiser criterion is not appropriate for determining the number of clusters. In response, we removed the reference to the Kaiser criterion and clarified the criteria used to select the cluster solution.  In the revised Analysis section, we clarified the methodological basis of the analytical pipeline. The use of Factor Analysis of Mixed Data (FAMD) implemented in FactoMineR is now explicitly grounded in Husson et al. (2016), and we also added Fermon et al. (2026) as a recent example of a comparable methodological approach.  Cluster selection is now described as being based on the hierarchical clustering dendrogram, changes in within-cluster inertia, cluster size and the interpretability of alternative solutions. We also added internal validation indices, including average silhouette width and gap statistics, and conducted stability checks using different random seeds and split-sample validation.  While these analyses did not identify a single unequivocal statistical optimum, they did support the three-cluster solution as offering the best compromise between validation indices, parsimony, cluster size and substantive interpretability.  These validation indices are reported in Supplementary Table A in a supplementary file. |
| **7.** Another major concern is the sampling imbalance and nested structure of the data. The number of respondents varied substantially across universities, which can strongly influence the clustering outcomes and subsequent interpretation. The authors should present the cluster membership distributions by university and country and assess whether the clusters are driven by a small number of large sites. Sensitivity analyses using weighting or stratified resampling should be performed to ensure that the results are not dominated by a few high-response universities. When reporting associations between cluster membership and variables such as sex, field, or university, the nested nature of the data should be considered, at least through a clear discussion of the implications and ideally through multilevel or robust inference approaches. | In the **‘Results’** section, before Table 4, the following sentence was added: Table 4 reports the participating institutions that were significantly associated with cluster membership and therefore contributed most to the interpretation of site-level patterns. Given the unequal distribution of respondents across universities, these associations should be interpreted cautiously as exploratory site-level patterns rather than as representative institutional, national, or regional differences. (Line 309).  A note on the table 4 is also added: ^¶^ Only institutions that are significantly associated with cluster membership are displayed. Due to the unequal distribution of respondents across universities, these results should be interpreted as exploratory site-level associations. (Line 324).  We add to the **‘results’** section, before table 5, the following sentence: “The highest proportion of students in the Autonomous cluster was observed in participating institutions located in Prague, Poznań, and Wrocław. The Networked supervised cluster was more prevalent in Oslo, Dortmund, Lille, and Liège, while the Regular supervised cluster was more prevalent in Teramo, Porto, and Rome.” (Line 329).  We add to the **‘discussion’** section: “Cluster membership was therefore significantly associated with the participating institution. However, given the uneven distribution of respondents across universities, these associations should be interpreted with caution as exploratory, site-level patterns rather than as representative of institutional, national or regional differences.” (Line 416).  In the **‘limitation’** section we added: “Furthermore, participants were nested within universities and countries; however, the present exploratory clustering approach did not formally model this multilevel structure. Consequently, the associations between cluster membership and variables such as sex, field of study, employment status or university should be interpreted with caution, as they may partly reflect site-specific recruitment dynamics or institutional contexts. Future studies should use more balanced sampling strategies and consider multilevel or robust inference approaches when examining whether these profiles remain stable across universities and countries.” (Line 486). | Page 16,  Results,  Lines 300 to 304.  Page 17,  Results,  Legend table 4,  Lines 314 to 316.  Page 18,  Results,  Lines 329 to 332.    Page 18,  Discussion,  Lines 417 to 420.  Page 28  Limitations,  Line 486 to 492, | We agree that the imbalance in the number of respondents across universities, coupled with the nested structure of the data, required clearer reporting and more cautious interpretation.  In the revised manuscript, we have clarified that Table 4 reports the institutions that participated and were significantly associated with cluster membership, and which therefore contributed most to the interpretation of site-level patterns. Due to the uneven distribution of respondents across universities, we explicitly state that these associations should be interpreted with caution as exploratory site-level patterns rather than as representative of institutional, national or regional differences.  We have also developed the Limitations section by acknowledging that participants were nested within universities and countries and that the present exploratory clustering approach did not formally model this multilevel structure. Consequently, associations between cluster membership and variables such as sex, field of study, employment status or university should be interpreted with caution as they may partly reflect site-specific recruitment dynamics or institutional contexts. Future studies should employ more balanced sampling strategies and consider multilevel or robust inference approaches when examining whether these profiles remain consistent across different universities and countries.  → See response for comment 4 and 5 to reviewer 2. |
| **8.** Statistical reporting also requires careful attention. Several reported chi-square values appear as decimals, suggesting that some reported values may represent effect sizes, such as Cramér’s V, rather than chi-square test statistics.  The authors should correct the labeling and report complete information for each test, including the test name, statistic, degrees of freedom, sample size, p-value, and effect size with interpretation.  Tables should clearly indicate what each statistic represents to avoid misinterpretation of the results. | New paragraph replacement in the **‘Results’** section: “Pearson’s chi-square tests were conducted to examine associations between cluster membership and categorical variables. Significant associations were found between cluster membership and sex, χ²(2, N = 2,165) = 17.54, p < .001, Cramér’s V = .09, indicating a small effect; field of study, χ²(12, N = 2,165) = 73.18, p < .001, Cramér’s V = .13, indicating a small effect; student employment, χ²(2, N = 2,165) = 50.63, p < .001, Cramér’s V = .15, indicating a small effect; and university, χ²(22, N = 2,165) = 1406.82, p < .001, Cramér’s V = .57, indicating a large effect.” | Page 18,  Results,  Lines 321 to 327. | We thank the reviewer for identifying this reporting issue. We corrected the statistical reporting in the Results section by distinguishing Pearson’s chi-square statistics from Cramér’s V effect sizes. The revised text now reports, for each categorical association, the test name, χ² statistic, degrees of freedom, sample size, p-value, Cramér’s V, and effect-size interpretation. |
| **9.** Finally, the interpretation should be more cautious and more tightly linked to the evidence. Cultural or regional explanations should not be overstated, particularly given the imbalance across universities and nested structure of the data. Broad East and West generalizations should be avoided unless supported by analyses that appropriately account for the site and country effects | All changes made in response to this comment are shown in orange in the revised manuscript.  In **abstract**: “Differences were also observed across participating institutions, underscoring the importance of considering contextual factors when designing PA promotion strategies.” (Line 22).  In **result**s: “The highest proportions of students in this cluster were observed in the participating institutions located in Poznan (71.4%, p < .001), Prague (56.5%, p < .001), and Wroclaw (44.3%, p < .001).” (Line 282 to 284).  “The participating institutions with the highest representation in this cluster included Oslo (86.7%), Dortmund (70.0%), Lille (57.7%), and Liège (55.1%).” (Line 292 to 293).  “The participating institutions with the highest representation in this cluster included Teramo (56.9%), Porto (55.0%), and Roma (54.2%).” (Line 296 to 298).  In **discussion**: “Our findings suggest that PA practices varied across the participating institutions.” (Line 416)  “Students from the participating universities located in Prague, Poznan, and Wroclaw were more represented in the Autonomous cluster (Cluster 1), whereas students from some other participating institutions were more represented in the supervised clusters.” (Line 420 to 423)  “Finally, Cluster 3 (“Regularly Supervised”) had a higher representation of students from participating universities in Teramo, Rome, and Porto, indicating a greater involvement in organized activities with peers during the academic year.” (Line 427 to 430)  “While these regional effects should be interpreted with caution, these studies provide useful contextual background for interpreting differences between participating institutions. However, the current study was not designed to test cultural or regional mechanisms directly, and the observed patterns should therefore be interpreted as exploratory associations rather than causal evidence of broader regional differences. Future research using balanced samples across institutions and countries, together with multilevel designs, is needed to examine whether the observed profiles remain stable across different institutional and geographic contexts.” (Line 439 to 445). |  | All changes made in response to this comment are shown in orange in the revised manuscript. As Reviewers 1 and 2 raised similar concerns, we revised the manuscript throughout to avoid overinterpretation of regional, cultural, and university-level patterns. Orange text indicates all modifications made to temper these interpretations and to clarify that the findings should be understood as exploratory institutional-level associations rather than representative regional or cultural differences. |
| **10.** In terms of presentation, the manuscript would benefit from careful English editing to improve the clarity, grammar, and consistency of terminology. |  |  | We thank the reviewer for this comment. The manuscript has been carefully revised to ensure clarity, readability and consistency of terminology, and to improve the English grammar. A fluent English-speaking colleague also reviewed the revised version for language. Key terms have been standardised throughout the manuscript, including 'PA participation', 'sport participation', 'autonomous/self-organised practice', 'supervised practice', and 'institution/country-level descriptors'. |
| **11.** All variables used in the clustering should be clearly defined, including question wording, response scales, and coding decisions, either in the main text or in the supplementary materials. | **A supplementary file was added.** | “Supplementary file: explanatory notes for tables” | We thank the reviewer for this helpful suggestion. In order to improve transparency and interpretability, we have added a supplementary file which provides explanatory notes for each table, as well as a detailed description of the variables used in the clustering analysis. This supplementary file defines each variable, its ecological or analytical dimension, type, response/coding information and role in the analysis.  → Same comment of reviewer 2 (comment **11**) |
| **12.** The interpretability of the clusters would be strengthened by adding a concise summary table that highlights the most discriminative variables for each cluster with effect sizes or other discriminative statistics and a brief practical interpretation of the table. | To improve the interpretability of the cluster solution, we added the following sentence to the **'Results'** section: “Table 6 summarizes the main variables that discriminate the three cluster profiles. This should be read alongside the detailed statistical results reported in Tables 4 and 5”  Table 6 in full was added after Table 5 (page 20-21-22 and 23, line 349 to 357). | Page 18;  Results,  Lines 334 to 336.  Page 20-21-22 and 23,  Results, table 6  Lines 349 to 357. | We thank the reviewer for this helpful suggestion. To improve the interpretability of the cluster solution, we have added a concise summary table (table 6) that highlights the most discriminative variables for each cluster. |

**Revisions in Response to Comments from Reviewer 2:**

| ***Comment/feedback from peer review:***  In this column, the reviewer’s feedback verbatim. | ***Author(s) change:***  In this column, the change verbatim. | ***Location:***  In this column, the line number(s) and page of the author(s) change. | ***Additional author(s) notes about the change*** |
| --- | --- | --- | --- |
| **1.** I would like to thank for the opportunity to review this manuscript. Please see the following comments to consider to further increase the quality of this manuscript. This manuscript presents a large-scale, cross-national investigation of physical activity (PA) participation patterns among undergraduate students across 16 universities in 12 European countries. The study addresses a relevant public health issue—declining PA in emerging adulthood—and adopts an ecological perspective combined with cluster analysis of mixed data to identify typologies of engagement. |  |  | Thank you very much for your time, consideration, and constructive comments. We have taken them into consideration when revising the manuscript. |
| **2.** The manuscript states that it adopts an ecological model (pp. 12–14), but the operationalization of this model is not sufficiently explicit. While determinants at individual, social, environmental, and policy levels are mentioned, the analysis primarily focuses on behavioral patterns and contextual variables. | We have now clarified the following paragraph, in **‘introduction’** section: *“*In the present study, this ecological framework was employed both as a theoretical lens and as a means of organizing the variables included in the analysis. The individual-level factors included sex, age, field of study, employment status, and previous PA-related background. Social-level factors referred to the relational context of PA participation, such as practicing alone, with peers, in supervised settings, or with an animal. Environmental and organizational factors included the place of practice, timing, seasonality, duration, type of structure and equipment used. Finally, university and country indicators were considered as markers of the broader institutional and regional environments, although policy-level determinants were not measured directly.” (line 65) | Page 4,  Introduction,  Line 65 to 73. | We agree with the reviewer that the operationalisation of the ecological model required further clarification. In the revised manuscript, we have specified how the ecological framework informed the selection and organisation of variables. We now explicitly distinguish individual-level variables, social-context variables, environmental/contextual variables, and institutional/regional indicators. We also clarified that policy-level determinants were not directly measured in the present study and should therefore be interpreted as part of the broader contextual framing, rather than as directly tested variables. |
| **3.** The manuscript distinguishes between energetic definitions (Caspersen, 1985) and broader socio-cultural definitions (Piggin, 2020), yet the operationalization appears behaviorally categorical (e.g., supervised vs. autonomous; indoor vs. outdoor). | We added sentence in the **‘introduction’** section: “In this study, PA is conceptualized as a multidimensional phenomenon that includes energy expenditure as well as contextual, social, and experiential dimensions of participation. However, it is empirically operationalized based on observable behavioral and contextual indicators, such as autonomy, supervision, location, timing, social context, intensity, and equipment. This definition encompasses not only sports activities but also different forms of PA that can occur in organized and non-organized contexts.” (Line 78) | Page 4 and 5,  Introduction,  Line 78 to 83. | We have revised the manuscript to clarify the relationship between our conceptualisation of physical activity and its empirical operationalisation. Although we adopt a broad definition of physical activity that encompasses energetic, contextual, social and experiential dimensions (Caspersen et al., 1985; Piggin, 2020), the empirical measures concentrate on observable behavioural and contextual indicators of participation. These include autonomy, supervision, location, timing, social context, intensity and equipment. We have adjusted the manuscript to avoid overstating the socio-cultural scope of the operationalisation, and to align the conceptual framework more closely with the measured variables. |
| **4.** The sample is heavily skewed toward a few institutions (e.g., Prague: 24.1%; Rome & Teramo: 23.9%), while others contribute fewer than 3% of respondents (Table 2, pp. 16–17).  **5.** The manuscript notes that each university assisted in recruitment via institutional communication channels. This likely resulted in convenience sampling. | We added sentence in the **‘Materials and Methods’** section: “Participants were recruited via a convenience sampling strategy facilitated by partner universities. Of the 4,321 undergraduate students who responded to the survey, 2,165 participants (1,413 females) were retained in the final analytic sample after applying the inclusion criteria, complete-case selection, and data-screening procedures. Eligibility required participants to complete the questionnaire in full, be enrolled as first-year students in the HYPEM project, be aged between 18 and 25 years, declare truthful responses, and report no health conditions preventing participation in PA. The mean age of participants at the time of questionnaire completion was 20.02 years (±1.45). Detailed participant characteristics are summarised in Table 1.” (line 134)  We added sentence in the **‘Limitation**’ section: “Some universities contributed significantly more responses than others, which could potentially affect clustering. As participants were recruited through partner universities, with some institutions contributing substantially more than others, the sample should be considered a convenience sample rather than a representative sample of European undergraduates. Consequently, the clusters may partly reflect institution-specific recruitment dynamics. Therefore, the findings should be interpreted as exploratory rather than as representative estimates of European undergraduates.” | Page 7  Materials and Methods, Participants,  Line 134 to 142  Page 28  Limitations,  Line 477 to 483, | We agree with the reviewer that the uneven distribution of respondents across institutions is a significant limitation. We have revised the manuscript to explicitly describe the sampling strategy as a non-probabilistic convenience sample, recruited through institutionally mediated channels.  We further clarify that the findings should not be interpreted as representative of all European undergraduates, but rather as exploratory patterns observed within a large, diverse, cross-national sample. This limitation has been emphasised more strongly in the revised manuscript. |
| **6.** The two retained dimensions explain only 21.38% of total variance (p. 20). This is low for constructing typologies. | We add the following text on limitations : “In this exploratory framework, the proportion of explained variance is not a primary criterion of model adequacy; instead, the validity of the solution is grounded in the interpretability, stability, and substantive coherence of the derived clusters.” | Page 21  In “Limitations” – Ligne 410 | We thank the reviewer for this important methodological comment. We acknowledge that the proportion of variance explained by the two retained dimensions (21.38%) may appear modest. However, the aim of this analysis was the identification of heterogeneous patterns of physical activity participation using a clustering approach. In this context, the quality and interpretability of the resulting clusters are more relevant criteria than the proportion of explained variance.  The dimensionality reduction step was used primarily to facilitate clustering in a reduced feature space, while preserving the multivariate structure of the data. |
| **7.** The manuscript states that a three-cluster solution was retained based on scree plot and Kaiser criterion (p. 19). However the Kaiser criterion is typically used in factor analysis, not clustering.  **8.** Outliers were excluded using the Grubb test (p. 20), which is typically designed for univariate normal data. | We expanded the **Methods (analysis section)** section to provide further details on the outlier-screening procedure: “Prior to conducting the multivariate analyses, the dataset was screened for missing values, inconsistent responses and outliers. The extent and pattern of missing data were first examined. Participants with incomplete questionnaires or responses that did not meet the inclusion criteria were excluded from the multivariate analyses using a complete-case procedure. This resulted in a final analytic sample of N = 2,165 participants. Extreme values were examined at the univariate level for quantitative variables only, with cases being excluded when values were considered implausible or inconsistent with the study criteria. This screening process resulted in the exclusion of 124 observations. No multivariate outlier detection procedure was applied to the mixed dataset. To assess the robustness of the exclusion procedure, the clustering analysis was repeated with and without the excluded observations. The resulting cluster structure, composition and interpretation remained highly consistent.”  We also revised the **'Participants'** section to provide the following clarification: “Of the 4,321 undergraduate students who responded to the survey, 2,165 participants (1,413 females) were retained in the final analytic sample after applying the inclusion criteria, complete-case selection, and data-screening procedures.” | Page 11 and 12  Materials and methods,  Analysis,  Lines 197 to 207.  Page 7,  Materials and Methods, Participants,  Line 135 to 137. | We thank the reviewer for this important methodological comment. We agree that Grubbs’ test is designed for univariate outlier detection and should not be presented as a multivariate outlier procedure. In the revised manuscript, we have clarified that data screening was conducted prior to the multivariate analyses and concerned missing values, inconsistent responses, and extreme values at the univariate level for quantitative variables only.  We also clarified that no multivariate outlier detection procedure was applied to the mixed dataset. The wording has been revised accordingly.  → Same response for comment 4 and 5 to reviewer 1. |
| **9.** The claim that Eastern European students prefer more autonomous practice while Western /Southern students prefer organized practice (pp. 28–29) is intriguing but potentially overinterpreted. | All changes made in response to this comment are shown in orange in the revised manuscript. |  | All changes made in response to this comment are shown in orange in the revised manuscript. As Reviewers 1 and 2 raised similar concerns, we revised the manuscript throughout to avoid overinterpretation of regional, cultural, and university-level patterns. Orange text indicates all modifications made to temper these interpretations and to clarify that the findings should be understood as exploratory institutional-level associations rather than representative regional or cultural differences. |
| **10.** The manuscript concludes that women are more represented in the “Autonomous” cluster and suggests potential discomfort in gyms (pp. 29–30). | The discussion “Physical activity mode and sex” (line 396) has been modified  From : " These findings align with our observations and may offer insights into why some women may not feel entirely at ease in gym environment, alone or in structured group activities.”  To: “However, the present study did not directly assess perceived comfort, safety, belonging, or barriers related to structured or indoor PA settings. Therefore, the overrepresentation of women in the ‘Autonomous’ cluster (cluster 1) should not be interpreted as evidence of discomfort in structured or indoor PA settings. Rather, it suggests that flexible, self-organised, and less constrained forms of PA may be particularly relevant to consider when designing inclusive PA opportunities for undergraduate students.” (Line 456) | Page 27,  Discussion,  Line 456 to 461. | We agree with the reviewer that our interpretation of the possible discomfort experienced by women in gym environments was too speculative. The revised text now states that the overrepresentation of women in the Autonomous cluster should be interpreted as an association between sex and modes of physical activity participation in this sample. We also contextualise this result by referencing previous literature which shows that the PA participation of girls and women (including the associated barriers and facilitators) may be influenced by gender norms, social support, perceived safety, social identity and physical self-concept. However, we explicitly acknowledge that these mechanisms were not measured directly in the present study and that they should be investigated in future research. |
| **11.** However: “Use of ICT” is treated as a key profiling variable, but the construct is insufficiently described. | A supplementary file was added.  Legend of the table 3, 4 and 5: “Full variable definitions, response scales and coding decisions are provided in Supplementary File.” | **“Supplementary file: explanatory notes for tables”**  Page 14,  Results, table 3  Line 253 to 254.  Page 17,  Results, table 4  Line 311 to 312.  Page 14,  Results, table 5  Line 339 to 344. | We added a more precise definition of ICT use in the legend of Table 5, distinguishing between ICT communication and functional ICT use.  In addition, we added a supplementary material providing detailed definitions of all variables used in the clustering analysis, including ICT-related variables, their response/coding information, and their role in the interpretation of the cluster profiles.  → Same comment of reviewer 1 (comment **11**) |
