# Supplementary material for: Where, when, how, and with whom do young university students engage in physical activity? A cluster-derived spectrum of patterns across 16 European universities
Source: PLoS One. 2026 Nov 4;21(11):e0360751. doi: 10.1371/journal.pone.0360751 (PMC13734477; doi:10.1371/journal.pone.0360751)
Supplement: Supplementary file 4 [file pone.0360751.s005.docx]

Title of manuscript: **Where, when, how, and with whom do young university students engage in physical activity? A cluster-derived spectrum of patterns across 16 European universities.**

Dear Professor Emily Chenette,

We sincerely thank you for your careful assessment of our revised manuscript. We are grateful for your positive evaluation of the substantial improvements made during the previous revision, as well as for recognizing that the manuscript is now technically sound and largely meets the scientific criteria of PLOS ONE.

In response to the remaining comments, we have carefully revised the manuscript and addressed each point in detail. We harmonized the Data Availability Statement across all submission files, which now clearly specifies the availability of the anonymized dataset, variable dictionary, and R analysis scripts in Recherche Data Gouv : <https://doi.org/10.57745/HKG3H8>.

Particular attention was given to moderating the interpretation of cultural and geographical differences. We reviewed the relevant sections of the abstract, introduction, discussion, limitations, and conclusion to ensure consistency throughout the manuscript. Statements suggesting broader cultural, geographical, national, or regional influences were removed or reformulated. The findings are now consistently described in terms of contextual characteristics and exploratory differences observed across participating institutions. We substantially revised the conclusion to ensure that the interpretation remains fully aligned with the study design and does not extend beyond what the data support.

Finally, we conducted a thorough review of the language and formatting. Any remaining grammatical, syntactic, or typographical errors were corrected, and the terminology was standardized throughout the manuscript and the supporting information.

All changes made are highlighted in purple in the revised manuscript.

We believe these revisions further improve the manuscript's clarity, consistency, and interpretive accuracy. We hope that the revised version fully addresses the remaining concerns and is suitable for publication in PLOS ONE.

Thank you again for the opportunity to revise and resubmit our work.

Yours sincerely,

The Authors

Date of resubmission: 11 July 2026

**Revisions in Response to Comments from Reviewers:**

| ***Comment/feedback from peer review:***  In this column, please state the reviewer’s feedback verbatim. | ***Author(s) change:***  In this column, please include your change verbatim. | ***Location:***  In this column, please provide the line number(s) and page of the author(s) change. | ***Additional author(s) notes about the change*** |
| --- | --- | --- | --- |
| First, the Data Availability Statement must be made fully consistent across all manuscript files. The current version contains conflicting information (e.g., stating that data are within the manuscript while also referring to a repository deposit with a DOI). This must be clarified and aligned with PLOS ONE data policy requirements. Please also ensure that the availability of the analysis code is clearly specified. | **Data Availability Statement: The anonymised dataset, variable dictionary and R analysis scripts that underlie the findings reported in this study are publicly available on Recherche Data Gouv (DOI: https://doi.org/10.57745/HKG3H8).** | Title page, p. 2, currently lines 43–44.  (File: PONE-D-25-68206_title page within Revised manuscript) | Thank you. We have now made the necessary changes. The revised statement identifies the data repository and persistent DOI, and explicitly specifies the availability of the R analysis scripts. The wording has been harmonised across all submission materials. |
| Second, there is an apparent inconsistency in the reported data collection period (‘September 2022 to January 2022’), which should be corrected. | **The sentence was corrected as follows:  Data collection occurred between September 2021 and January 2023.** | Materials and Methods – Data collection procedure | Thank you for identifying this typographical error. The correct data collection period was September 2021 to January 2023. The date was corrected and checked throughout the manuscript. |
| Third, please ensure full consistency in methodological terminology across the manuscript and supplementary materials (e.g., FAMD versus PCA). | The manuscript, Supporting Information and Supplementary file_table_exaplanations were reviewed to ensure consistent use of the term Factor Analysis of Mixed Data (FAMD).  **The Results section was revised as follows:**  “Table 3 presents the significant correlations between the variables and dimensions identified through Factor Analysis of Mixed Data (FAMD).”  **The title of Table 3 was revised as follows:**  “Table 3. Significant correlations between variables and dimensions identified through Factor Analysis of Mixed Data (FAMD).”  **The S3 Table description was revised as follows:**  “S3 **Table 3. Significant correlations between variables and dimensions identified through factorial analysis of mixed data (FAMD).**  Full variable definitions, response scales, and coding decisions are provided in the Supplementary File.”  In ‘Supplementary file_table_exaplanations’ – Overview of supplementary tables (p. 10)  S3 **Table 3. Significant correlations between variables and dimensions identified through factorial analysis of mixed data (FAMD).**  (p. 03)  S3 – **Table 3. Significant correlations between variables and dimensions identified through factorial analysis of mixed data (FAMD).** |  | Thank you for highlighting this remaining inconsistency. We reviewed the Analysis and Results sections, all table titles, the Supporting Information, and the explanatory notes in the Supplementary File. |
| Fourth, while the interpretation has been substantially improved, some statements, particularly in the Conclusion, still suggest broader cultural or geographical influence than can be supported by the study design. These should be further moderated for internal consistency. | **All of the changes are marked in purple in the manuscript (Abstract, Discussion, Limitations, Conclusion)** |  | Thank you for this important comment. We carefully reviewed the manuscript and revised all passages that suggested broader cultural or geographical influences than the study design could support. All changes made in response to your comment are highlighted in purple in the revised manuscript.  More specifically, we replaced terminology referring to sociocultural influences, cultural factors, geographical variations, or regional effects with more cautious wording focused on contextual characteristics, associations, and observed differences across participating institutions. In the abstract and study objectives, causal expressions such as “influence” and “determinant” were replaced with association-based terminology.  In the Discussion, the subsection title was changed from “Physical activity and cultural differences” to “Physical activity and differences across participating institutions,” and the interpretation of institution- and sex-related patterns was further moderated.  The Limitations section was streamlined to clarify the implications of the convenience-based recruitment strategy, the unequal sample sizes of the institutions, and the nested structure of the data while avoiding repetitive statements.  Finally, the conclusion was substantially revised. References to the "influence of cultural factors," "geographical and cultural variations," and the comparison between participation profiles and cultural factors were removed. The revised conclusion now focuses on the diversity of PA participation profiles, the conditions in which PA is practiced, and the differences observed across the participating institutions.  These revisions were made to ensure consistency in interpretation throughout the manuscript and to ensure that it does not extend beyond what can be supported by the present study. |
