# Supplementary material for: Where, when, how, and with whom do young university students engage in physical activity? A cluster-derived spectrum of patterns across 16 European universities
Source: PLoS One. 2026 Nov 4;21(11):e0360751. doi: 10.1371/journal.pone.0360751 (PMC13734477; doi:10.1371/journal.pone.0360751)
Supplement: Supplementary file 5 [file pone.0360751.s006.docx]

Title of manuscript: **Where, when, how, and with whom do young university students engage in physical activity? A cluster-derived spectrum of patterns across 16 European universities.**

**Revisions in response to comments from the academic editor:**

| ***Comment/feedback from peer review:*** | ***Author(s) change:*** | ***Location*** | ***Additional author(s) notes about the change*** |
| --- | --- | --- | --- |
| **Data Availability Statement:** The clean title page still states that “All relevant data are within the manuscript,” whereas the main manuscript and submission metadata identify the Recherche Data Gouv repository. Please replace the title-page statement with the harmonised repository statement and verify that the DOI provides access to the anonymised dataset, data dictionary, and R scripts in accordance with PLOS ONE policy. | **Data availability statement.** The anonymized dataset, variable dictionary, and R analysis scripts underlying the findings reported in this study are publicly available in Recherche Data Gouv (DOI: <https://doi.org/10.57745/HKG3H8>).  Title page, page 02, lines 43 to 45.  **Conflict of interest disclosure.** The authors report there are no competing interests to declare.  **Acknowledgments.** The authors thank all the universities involved in the project and all participants who contributed to the study. | **Title page:** p. 2, former line 43-45.  **End of manuscript:** p. 34, former lines 611 to 613. | We replaced the statement on the clean title page that 'All relevant data are within the manuscript' with a statement that consistently refers to the Recherche Data Gouv repository and the corresponding DOI.  The anonymised dataset, variable dictionary and R analysis scripts have all been deposited in the Recherche Data Gouv repository under the following DOI: https://doi.org/10.57745/HKG3H8. At the time of resubmission, the anonymised dataset and variable dictionary had been validated by the University of Lille and the repository platform, and were visible in the published repository record. The R analysis script had also been uploaded to the same repository, as documented in the deposit confirmation provided as an attachment to this response. However, it is still awaiting institutional and repository validation, meaning it is not yet visible through the public DOI record.  We acknowledge that access to all three materials cannot yet be fully verified through the DOI. We are following up with the University of Lille repository team to ensure that the R script is validated, and that the anonymised dataset, variable dictionary and R analysis scripts are accessible via the same DOI, before publication.  |
| **Participant flow:** Please provide a transparent breakdown of exclusions from the 4,321 initial respondents to the 2,165 participants included in the final analysis. Report the number excluded for each eligibility criterion, incomplete data, inconsistent responses, health-related exclusions, and outlier screening. Where feasible, provide a brief comparison of included and excluded respondents or explain why this could not be performed. | **Materials and methods: Participants**: Participants were recruited through convenience sampling at partner universities. Of the 4,321 individuals who initiated the survey, 1,061 did not complete any questionnaire items and were excluded from further analyses,. The remaining 3,260 underwent eligibility and data-completeness screening.  Eligibility criteria included enrolment at a participating university, age between 18 and 25 years, confirmation of response truthfulness, and absence of a health condition preventing participation in physical activity. Before the multivariate analyses, 3,260 individuals who provided questionnaire data were screened sequentially for eligibility and data completeness. Individuals were excluded because of missing age (n = 890), age outside the eligible range of 18–25 years (n = 80), missing year-of-study information (n = 1), or missing information on their participating university (n = 124). No participants were excluded based on self-reported dishonesty, health-related exclusion criteria, logically inconsistent responses (e.g., contradictory responses across questionnaire items), or outlier screening (n = 0 for each). The final analytic sample comprised 2,165 participants. All values were checked against the predefined response options, and no multivariate outlier-detection procedure was applied to the mixed dataset. Their mean age was 20.02 ± 1.45 years. Detailed participant characteristics are presented in Table 1.  **Analysis**: Associations between cluster membership and categorical supplementary variables were additionally examined using Pearson’s chi-square tests, with Cramér’s V reported as a measure of association strength. Descriptive statistics included means, standard deviations, frequencies, and proportions, as appropriate.  (…)  in R version 4.3.0 using packages including FactoMineR, factoextra, cluster, psych, rstatix, and effectsize. | **Participants:** p. 9, lines **182 to 199.**  **Analysis and exclusions:** p. 13, lines **243-252,** and  p. 13, lines **254-284.** | We have clarified the distinction between survey initiation and the provision of questionnaire data, and we now provide a complete, sequential breakdown of exclusions.  Of the 4,321 individuals who initiated the online survey, 1,061 did not complete any questionnaire items and were excluded before eligibility and data-completeness screening.  Of the remaining 3,260 individuals, exclusions were applied sequentially for missing age data (n = 890), being outside the eligible age range of 18–25 years (n = 80), missing year-of-study information (n = 1) and missing information on the participating university (n = 124). This resulted in a final analytical sample of 2,165 participants.  Confirmation of truthfulness of responses and absence of health conditions preventing participation in physical activity were also assessed as eligibility criteria. However, these criteria did not result in any additional exclusions, as all individuals who reached this stage met them (n = 0 for each criterion). Similarly, no individuals were excluded due to inconsistent responses or statistical outlier screening (n = 0 for each). We removed previous statements indicating that 124 observations had been excluded as outliers and that the clustering analysis had been repeated with and without the excluded observations, as these statements did not accurately describe the participant selection procedure.  A formal comparison of the entire included and excluded groups could not be performed reliably. Of the excluded individuals, 1,061 provided no questionnaire data, 890 did not provide age information, and other sociodemographic characteristics were unavailable or incomplete for many excluded records. Consequently, there was no consistent set of variables on which to base a comparison of all included and excluded individuals. Furthermore, a comparison limited to the subset with available data could have been misleading.  All changes have been made in the Participants and Analysis section.  As part of this revision, we also reviewed the analysis section to improve consistency between the statistical procedures described in the methods section and those reported in the results section. Pearson’s chi-square tests and Cramér’s V were previously added to the Results section in response to a request from an earlier reviewer, but these analyses were not explicitly reported in the Methods section. This has now been corrected by specifying that associations between cluster membership and supplementary categorical variables were examined using Pearson’s chi-square tests, with Cramér’s V reported as a measure of association strength. These additions do not reflect new analyses, but rather ensure that all procedures reported in the 'Results' section are fully described and reproducible in the 'Methods' section. |
| **Interpretation of sex-related findings:** Please remove or substantially qualify the recommendation for women-only spaces. The study did not assess perceived safety, comfort, belonging, or the effectiveness of sex-specific interventions, and the observed association between sex and cluster membership was small. Such proposals should be presented as questions for future research rather than evidence-based recommendations from the present study. | Discussion: “Physical activity mode and sex”:  Instead, this pattern raises the question of whether flexible, self-organised, or less constrained PA formats are perceived differently by women and men, which should be examined directly in future research.  **Conclusion:**  The findings also reveal significant sex differences in PA engagement. These results do not support recommendations for a specific sex-targeted PA format. Future research could examine whether organisational arrangements such as smaller group sizes, outdoor settings, or other activity formats are perceived differently by women and men and whether they are associated with participation, comfort, or belonging. | **Sex effect in Results:** p. 20, lines **367 to 373**.  **Discussion, “Physical activity mode and sex”:** p. 29, lines **497 to 518**, and p. 30, lines **519 to 522**.  **Conclusion:** p. 32, lines **580-587**.  **Table 4 sex categories:** p. **18,19** | We revised the interpretation of the sex-related findings throughout the Discussion and Conclusion.  The terminology was also standardized as **sex**, **women**, and **men**, rather than alternating between sex and gender. |
| **Intervention and effectiveness language:** Please revise statements suggesting that particular PA formats are “effective,” will “enhance” activity levels, or will produce healthier routines. The cross-sectional exploratory design identifies participation profiles and associations but does not evaluate intervention effectiveness or changes in PA. Implications should be framed as hypotheses or considerations for future prospective and intervention research. | The final sentence of the **Abstract** was replaced with: **“**Differences were also observed across participating institutions, indicating that contextual factors should be considered when interpreting these exploratory profiles. These profiles may help generate hypotheses about how university PA opportunities could be designed and evaluated in future prospective and intervention studies.**”**  **Introduction:**  In this context, describing students’ PA and sport participation patterns and the factors associated with them may help formulate questions for future research on university PA promotion.  Understanding how these factors are associated with students’ PA and sport behaviours may help formulate questions for future research. Examining students’ reported locations, timing, social contexts, and forms of PA can provide a broader description of participation patterns and inform the design of prospective and intervention studies.  “less research has specifically examined how engagement in PA varies according to contextual factors”  By identifying the most salient patterns of PA participation, this analysis may generate hypotheses about how university PA opportunities could be designed and evaluated in future research.  **Results:**  In Table 6, we renamed the final column “Considerations for future research” and reformulated the cluster-specific implications as testable hypotheses, replacing prescriptive statements about what university PA programmes may need to provide with suggestions for future studies to examine the acceptability, uptake, and association of these formats with continued participation.  Discussion: “The evolution…”:  This finding is noteworthy because it shows that similar levels of PA may involve diverse participation formats. Recent reports and studies have highlighted the prominence of less structured, home-based, and digitally supported forms of PA among young adults [4,30]. Consistent with this literature, the profiles identified in the present study included home-based, pet-related, digitally supported, and free-of-charge practices. Considering the temporal, spatial, social, and equipment-related conditions of participation may therefore complement analyses focused primarily on the quantity of PA.  Discussion: “Physical activity in new ways”:  Previous research suggests that flexible PA formats may be associated with more personalised or enjoyable experiences and with continued participation [35]; however, these outcomes were not evaluated in the present study. Structured activities provide opportunities for social interaction and organised routines, whereas unstructured formats may be more compatible with independence and individual scheduling preferences. The coexistence of supervised and unsupervised participation profiles suggests a need to examine whether PA opportunities adapted to different preferences influence uptake, enjoyment, or continued participation.  Given that the transition to higher education represents a critical life stage for PA participation [8,10], the varied participation profiles identified in this study suggest that inclusive programmes accommodating different forms of PA warrant consideration and prospective evaluation.  Discussion: “Physical activity mode and sex”:  Future research could examine whether students perceive PA sessions organised in smaller groups, outdoor settings, or other formats differently, and whether these formats are associated with enjoyment, uptake, or continued participation. As individual preferences vary widely [30], the effects of such organisational arrangements should be evaluated rather than assumed. Prospective and intervention studies are also needed to test whether the experiential and pedagogical characteristics of structured activities influence these outcomes [39]. Given the small association between sex and cluster membership, it would be unwise to assume that any particular format is preferable or effective for women or men.  All the ‘Implications for future research and practice’ change.  **Conclusion**  This study illuminates the varied landscape of PA engagement among undergraduate students at participating European universities. By identifying three distinct clusters: ‘Autonomous,’ ‘Networked Supervised,’ and ‘Regularly Supervised’, the findings underscore the importance of considering not only the quantity and intensity of PA, but also how, where, when, and under what social and organisational conditions PA is practised. Our results contribute to the existing body of knowledge by illustrating the dynamic nature of PA preferences among undergraduates. Tailored approaches, such as offering diverse activity options and reimagining PA and sports pathways, **may warrant consideration and evaluation in future research as ways of responding to students’ varied behaviours and preferences**. Universities **could examine whether creating more inclusive and flexible opportunities that align with students’ lifestyles is associated with greater engagement or healthier routines**. Additionally, the differences observed across participating institutions **suggest that the relevance of flexible and context-sensitive approaches should be examined prospectively**. **These exploratory findings do not support a single approach to promoting PA participation**. Instead, a nuanced understanding of students’ preferences and needs **may help formulate hypotheses for prospective and intervention studies conducted during the transition from secondary school to higher education**. The findings also reveal sex differences in PA engagement. These results do not support recommendations for a specific sex-targeted PA format. Future research could examine whether organisational arrangements such as smaller group sizes, outdoor settings, or other activity formats are perceived differently by women and men and whether they are associated with participation, comfort, or belonging. Furthermore, the study underscores the importance of considering how PA is experienced and the conditions under which participation occurs when examining undergraduate students’ preferences. The acceptability and potential effects of offering varied exercise formats and opportunities for autonomous participation should be evaluated in future prospective and intervention studies. These insights may help generate hypotheses and inform the design and evaluation of future university initiatives and public policies aimed at reducing sedentary lifestyles. Finally, investigating PA based on the types of experiences and practices undergraduate students seek provides a broader framework for understanding participation beyond quantity and intensity alone. Whether offering PA in varied formats increases students’ PA levels, supports continued participation, or contributes to healthier routines should be tested through prospective and intervention research. | **Abstract:** p. 3, lines **72 to 73**, and p. 4, lines **74 to 75**.    **Introduction:** p. 6, lines **107 to 109**; p. 8, lines **159 to 162**; p. 8, lines **166 to 169**; p. 8, line **177**, to p. 9, line **179**.  **Table 6 caption:** p. 22  **Table 6 content:** p. **22 to 24**  **Table 6 explanatory note:** p. 25  **Discussion:** p. 26, lines **423 to 429**; p. 27, lines **450 to 456** and **461 to 464**.  **Discussion, sex:** p. 29, lines **515 to 518**, and p. 30, lines **519 to 522**.  **Implications** p. 31.  **Conclusion:** p. 32, lines **565 to 587**, and p. 33, lines **588 to 599**. | We systematically reviewed the Abstract, Introduction, Results interpretation, Discussion, implications section, and Conclusion to remove or qualify causal, prescriptive, and effectiveness-related language.  The revised manuscript consistently distinguishes the cross-sectional profiles and associations observed in this study from intervention effects that require prospective or experimental evaluation.  No new analyses were performed for this revision. |
| **Ethics statement:** Please ensure that the ethics statement appears only in the Methods section, in accordance with the journal’s previous instruction, and remove the redundant statement from the title page if required. | The ethics statement was removed from the title page, as requested, and is now included exclusively in the Methods section. | **Deleted from title page:** page 02, former lines 45–46.  **Retained in Materials and Methods, Data collection procedure:** p 12, lines 230 to 242. | No ethics statement remains on the title page. The complete approval and informed-consent information appear only in the Methods section as its asked. |
| **Final consistency check:** Please perform one final proofread to ensure that cluster labels, percentages, institutional names, funding statements, and supplementary-file references are identical across the clean manuscript, title page, tracked version, response letter, and submission metadata. | The three cluster labels and their associated percentages were standardized throughout as “Autonomous” (42.6%), “Networked Supervised” (28.6%), and “Regularly Supervised” (28.8%).  Capitalization was harmonized in the Abstract, Results headings, Tables 4–6, Discussion, Conclusion, and Supporting Information.  All references to the explanatory supplementary document were standardized as “Supplementary file,” with a lower-case “f,” in the manuscript, Supporting Information, and the title of the supplementary document.  Author and institutional names were also checked and corrected, including the spellings of Alexis Mauger, Barbara Mazza, Tobias Vogt, and “University of Teramo, Teramo, Italy.”  The names of all participating institutions were checked and standardized throughout the affiliation list, Tables 2 and 4, the Results and Discussion sections, the Supporting Information. Institutional designations, spelling, location names, and diacritics were harmonized consistently across all submission files.  In accordance with the journal’s previous instruction, funding-related text was removed from the manuscript files and retained only in the Financial Disclosure section of the submission system.  These corrections were applied consistently across the clean manuscript, tracked manuscript, response letter, supporting files, and submission metadata. | **All the changes are in all the manuscript in orange.**  **Authors and affiliations:** p. 1, lines **2 to 25**, and p. 2, lines **26 to 34**.  **Cluster labels in Abstract,**  **in Results.**  **Tables 4 and 6:** p. **18**.  **Discussion:** p. 25, lines **406 to 409**.  **Conclusion:** p. 32, lines **565 to 569**.  **Institution names in Table 2, Results, Discussion.**  **Supporting Information:** p. 38, lines **716 to 722**; p. 39, lines **723 to 745**; p. 40, lines **746 to 761**. | We conducted a final cross-document consistency check. Cluster labels and percentages, institutional and author names, supplementary-file terminology, and repeated statements were harmonised across the clean manuscript, tracked manuscript, response letter, supporting files.  These corrections concern nomenclature and reporting consistency and do not affect the reported results. |
| **Final consistency check:** funding statements |  | No funding statement remains in the manuscript. | Funding-related text was removed from the clean and tracked manuscripts, including the funding statement and the acknowledgment of the Lille I-Site research cluster. The complete funding information was retained and verified exclusively in the **Financial Disclosure** section of the online submission system, in accordance with the journal’s previous instruction. |

**Revisions in Response to Comments from Journal Requirements:**

| ***Comment/feedback from peer review:*** | ***Author(s) change:*** | ***Location:*** | ***Additional author(s) notes about the change*** |
| --- | --- | --- | --- |
| Please review your reference list to ensure that it is complete and correct. If you have cited papers that have been retracted, please include the rationale for doing so in the manuscript text, or remove these references and replace them with relevant current references. Any changes to the reference list should be mentioned in the rebuttal letter that accompanies your revised manuscript. If you need to cite a retracted article, indicate the article’s retracted status in the References list and also include a citation and full reference for the retraction notice. | The in-text citation supporting the discussion of structured PA programmes was corrected. The sentence now reads: “Prospective and intervention studies are also needed to examine whether the design and delivery characteristics of structured PA programmes influence these outcomes [10].”  No references were added to or removed from the reference list. | Discussion: p.30, line 519.  All references: pp. 34–38, lines 600–716. | We reviewed the complete reference list for bibliographic accuracy and retraction status. No retracted articles were identified. During this review, we identified that the previous citation [39] did not directly support the statement concerning the characteristics of structured PA programmes. We therefore revised the wording and replaced citation [39] with reference [10], a systematic review of controlled trials examining PA promotion among university students. The numbering and content of the reference list remain unchanged. |
