# Supplementary material for: Where, when, how, and with whom do young university students engage in physical activity? A cluster-derived spectrum of patterns across 16 European universities
Source: PLoS One. 2026 Nov 4;21(11):e0360751. doi: 10.1371/journal.pone.0360751 (PMC13734477; doi:10.1371/journal.pone.0360751)
Supplement: Supplementary file 6 [file pone.0360751.s007.docx]

Title of manuscript: **Where, when, how, and with whom do young university students engage in physical activity? A cluster-derived spectrum of patterns across 16 European universities.**

**Revisions in response to comments from the academic editor:**

| ***Comment/feedback from peer review:*** | ***Author(s) change:*** | ***Location*** | ***Additional author(s) notes about the change*** |
| --- | --- | --- | --- |
| The R analysis script must be publicly accessible through the stated repository DOI, or the Data Availability Statement must accurately reflect its current accessibility. |  |  | We have verified that the research materials deposited in the Recherche Data Gouv are accessible. We have checked with our university, and everything is accessible now.  DOI: <https://doi.org/10.57745/HKG3H8> |
| The denominators used for the percentages in Table 4 must be explicitly defined, particularly because several displayed rows do not sum to 100% across clusters. | **A clarification was added to the table 4 :** Percentages are calculated within each category and indicate the proportion of participants in that category assigned to each cluster. | Results, Table 4 and Table 4 note. | We have clarified how the percentages reported in Table 4 are calculated and presented. The percentages in each row are calculated within the relevant category and show how participants are distributed across the three clusters. During this review, we identified several inconsistencies in the previously reported percentages in Table 4. These values have been recalculated and corrected where necessary, and the corresponding percentages in the main text have been updated. All changes are highlighted in blue in the revised manuscript. We have also added a note below Table 4. The underlying clustering solution and statistical analyses were not altered. We thank the Editor for bringing this issue to our attention and apologise for the reporting inconsistencies. |
| **Sample terminology:**  The manuscript must clarify whether the sample comprised exclusively first-year students or undergraduate students from multiple academic years and use consistent terminology throughout. | Study Design was revised: “A cross-sectional design was employed to collect data from undergraduate students across Europe at a single point in time.” | **Line 157** | Thank you for highlighting this inconsistency. The study sample comprised undergraduate students, not just first-year students. The term 'first-year students' in the 'Study Design' section was inaccurate and inconsistent with the population described throughout the manuscript. We have therefore replaced it with 'undergraduate students' and reviewed the manuscript to ensure consistent terminology is used throughout. |
| **Exploratory interpretation:**  The statement that participation format and regularity differentiate students more strongly than quantity or intensity should be moderated to reflect the exploratory nature of the analysis. | **The sentence was revised:** The present study reveals distinct patterns and differences in how undergraduate students across European universities engage in PA. Two main categories were identified: 'Autonomous' and 'Supervised'. The latter category was further subdivided into 'Regularly Supervised' and 'Networked Supervised' groups. In this exploratory analysis, participation format and regularity emerged as salient dimensions for distinguishing these profiles, alongside quantity- and intensity-related characteristics. | **Discussion**  **Lines 361 to 366** | We have amended this statement to better reflect the exploratory nature of the analysis. Rather than stating that the format and regularity of participation differentiate students more strongly than the quantity or intensity of participation, we now indicate that these characteristics, alongside quantity- and intensity-related characteristics, emerged as salient dimensions for distinguishing the identified profiles. |

**Revisions in Response to Comments from Journal Requirements:**

| ***Comment/feedback from peer review:*** | ***Author(s) change:*** | ***Location:*** | ***Additional author(s) notes about the change*** |
| --- | --- | --- | --- |
| **Journal Requirements**  **References:** Please review the reference list for completeness, accuracy, and retraction status. Any changes to the reference list should be mentioned in the rebuttal letter. | No references were added to or removed from the reference list. | Reference section. | We reviewed the complete reference list for bibliographic accuracy and retraction status. No retracted articles were identified. |
