# Supplementary material for: Where, when, how, and with whom do young university students engage in physical activity? A cluster-derived spectrum of patterns across 16 European universities
Source: PLoS One. 2026 Nov 4;21(11):e0360751. doi: 10.1371/journal.pone.0360751 (PMC13734477; doi:10.1371/journal.pone.0360751)
Supplement: Supplementary file 7 [file pone.0360751.s008.docx]

Title of manuscript: **Where, when, how, and with whom do young university students engage in physical activity? A cluster-derived spectrum of patterns across 16 European universities.**

**Revisions in response to comments from the academic editor:**

| ***Comment/feedback from peer review:*** | ***Author(s) change:*** | ***Location*** | ***Additional author(s) notes about the change*** |
| --- | --- | --- | --- |
| The combined proportion of the two supervised clusters is 57.4% rather than 67.4%. Please also ensure that the age range is reported consistently throughout the manuscript. | Nevertheless, conventional supervised activities remain highly popular, with 57.4% of surveyed students over-represented in this category. | *Discussion Lines 413 to 414* | Thank you for identifying these points.  We apologize for the numerical inconsistency regarding the combined proportion of the two supervised clusters. This was a reporting error in the Discussion. The value has now been corrected from **67.4% to 57.4%**, corresponding to the combined proportions of the Networked Supervised (28.6%) and Regularly Supervised (28.8%) clusters. |
| Please also ensure that the age range is reported consistently throughout the manuscript. | Young adults are particularly at risk of dropping out of physical activity (PA). European policies have addressed this issue by promoting PA and encouraging better monitoring of PA through standardized and specific measures of its quantity. The current paper used an ecological approach that considers interrelated determinants at the individual, social, environmental, and policy levels to survey 2,165 undergraduate students (65.3% female) aged 18-25 years from 16 universities via an online questionnaire as part of the “How Young People in Europe Move” (HYPEM) project. | **Abstract**  **Lines 6 to 11** | We also carefully reviewed the reporting of age throughout the manuscript. The study sample consistently comprised undergraduate students aged **18–25 years**. Other age ranges mentioned in the manuscript refer to findings from previously published studies and reports cited in the Introduction and Discussion, rather than to the age range of the present study sample. To avoid any potential ambiguity, we revised the Abstract so that the age range of the study sample is explicitly reported as **18–25 years.** |

**Revisions in Response to Comments from Journal Requirements:**

| ***Comment/feedback from peer review:*** | ***Author(s) change:*** | ***Location:*** | ***Additional author(s) notes about the change*** |
| --- | --- | --- | --- |
| **Journal Requirements**  **References:** Please review the reference list for completeness, accuracy, and retraction status. Any changes to the reference list should be mentioned in the rebuttal letter. | No references were added to or removed from the reference list. | Reference section. | We reviewed the complete reference list for bibliographic accuracy and retraction status. No retracted articles were identified. |
