# Supplementary material for: Where, when, how, and with whom do young university students engage in physical activity? A cluster-derived spectrum of patterns across 16 European universities
Source: PLoS One. 2026 Nov 4;21(11):e0360751. doi: 10.1371/journal.pone.0360751 (PMC13734477; doi:10.1371/journal.pone.0360751)
Supplement: Peer review history

# Peer review: Decision Letter 0

**DOI:** 10.1371/journal.pone.0360751.r001

**Authors:** Camelia Delcea

22 Feb 2026

PONE-D-25-68206

Where, When, How, and With Whom Do Young University Students Engage in Physical Activity? A Cluster-Derived Spectrum of Patterns Across 16 European Universities.

PLOS One?

Dear Dr. Llena,

Thank you for submitting your manuscript to PLOS ONE. After careful consideration, we feel that it has merit but does not fully meet PLOS ONE’s publication criteria as it currently stands. Therefore, we invite you to submit a revised version of the manuscript that addresses the points raised during the review process.

Both reviewers recognize the importance and scale of the study. However, substantial revisions are required to improve methodological transparency, reproducibility, cluster validation, sampling considerations, and clarity of theoretical framing. Strengthening these areas will significantly increase confidence in the robustness and interpretability of the findings.

Please submit your revised manuscript by Apr 08 2026 11:59PM. If you will need more time than this to complete your revisions, please reply to this message or contact the journal office at plosone@plos.org. When you're ready to submit your revision, log on to https://www.editorialmanager.com/pone/ and select the 'Submissions Needing Revision' folder to locate your manuscript file.

- A letter that responds to each point raised by the academic editor and reviewer(s). You should upload this letter as a separate file labeled 'Response to Reviewers'.
- A marked-up copy of your manuscript that highlights changes made to the original version. You should upload this as a separate file labeled 'Revised Manuscript with Track Changes'.
- An unmarked version of your revised paper without tracked changes. You should upload this as a separate file labeled 'Manuscript'.

If you would like to make changes to your financial disclosure, please include your updated statement in your cover letter. Guidelines for resubmitting your figure files are available below the reviewer comments at the end of this letter.

If applicable, we recommend that you deposit your laboratory protocols in protocols.io to enhance the reproducibility of your results. Protocols.io assigns your protocol its own identifier (DOI) so that it can be cited independently in the future. For instructions see: https://journals.plos.org/plosone/s/submission-guidelines#loc-laboratory-protocols. Additionally, PLOS ONE offers an option for publishing peer-reviewed Lab Protocol articles, which describe protocols hosted on protocols.io. Read more information on sharing protocols at https://plos.org/protocols?utm_medium=editorial-email&utm_source=authorletters&utm_campaign=protocols.

We look forward to receiving your revised manuscript.

Kind regards,

Camelia Delcea

Academic Editor

PLOS One

Journal Requirements:

When submitting your revision, we need you to address these additional requirements.

1. Please ensure that your manuscript meets PLOS ONE's style requirements, including those for file naming. The PLOS ONE style templates can be found at

https://journals.plos.org/plosone/s/file?id=wjVg/PLOSOne_formatting_sample_main_body.pdf and

https://journals.plos.org/plosone/s/file?id=ba62/PLOSOne_formatting_sample_title_authors_affiliations.pdf

2. When completing the data availability statement of the submission form, you indicated that you will make your data available on acceptance. We strongly recommend all authors decide on a data sharing plan before acceptance, as the process can be lengthy and hold up publication timelines. Please note that, though access restrictions are acceptable now, your entire data will need to be made freely accessible if your manuscript is accepted for publication. This policy applies to all data except where public deposition would breach compliance with the protocol approved by your research ethics board. If you are unable to adhere to our open data policy, please kindly revise your statement to explain your reasoning and we will seek the editor's input on an exemption. Please be assured that, once you have provided your new statement, the assessment of your exemption will not hold up the peer review process.

3. We note that the grant information you provided in the ‘Funding Information’ and ‘Financial Disclosure’ sections do not match.

When you resubmit, please ensure that you provide the correct grant numbers for the awards you received for your study in the ‘Funding Information’ section.

4.  Your ethics statement should only appear in the Methods section of your manuscript. If your ethics statement is written in any section besides the Methods, please delete it from any other section.

5. If the reviewer comments include a recommendation to cite specific previously published works, please review and evaluate these publications to determine whether they are relevant and should be cited. There is no requirement to cite these works unless the editor has indicated otherwise.

[Note: HTML markup is below. Please do not edit.]

Reviewers' comments:

Reviewer's Responses to Questions

**Comments to the Author**

1. Is the manuscript technically sound, and do the data support the conclusions?

Reviewer #1: Partly

Reviewer #2: Yes

**********

2. Has the statistical analysis been performed appropriately and rigorously?

Reviewer #1: Yes

Reviewer #2: Yes

**********

3. Have the authors made all data underlying the findings in their manuscript fully available?

The PLOS Data policy

Reviewer #1: No

Reviewer #2: Yes

**********

4. Is the manuscript presented in an intelligible fashion and written in standard English?

Reviewer #1: No

Reviewer #2: Yes

**********

Reviewer #1: Thank you for the opportunity to review this manuscript, which examines where, when, how, and with whom first-year university students engage in physical activity across 16 European universities. The cross-national scope and intention to characterize patterns beyond simple participation levels are valuable. However, I recommend major revisions because the key elements required for technical clarity, reproducibility, and compliance with the PLOS ONE policy have not been adequately addressed, and several reporting and methodological issues currently limit confidence in the conclusions.

A central requirement that must be strengthened is the availability and reproducibility of data. The current information on data availability does not clearly specify a concrete public location for the dataset underlying the analyses, such as a repository link, a DOI, or an accession number. To meet PLOS ONE requirements, the authors should deposit the complete de-identified dataset and an accompanying data dictionary or codebook in an appropriate public repository, provide a stable URL, DOI, or accession number in the Data Availability Statement, and share the analysis code needed to reproduce the dimensionality reduction and clustering workflow, as well as the reported tables and figures. If there are legitimate restrictions on sharing data due to privacy or third-party conditions, these restrictions should be explicitly stated, and the minimum necessary shareable dataset and documentation should still be provided.

The manuscript also requires clearer and more consistent ethical reporting. The ethics information appears inconsistent regarding the name of the approving ethics committee. The authors should ensure that the approving body is correctly and consistently identified throughout the manuscript and the supporting documents. In addition, it should be clarified whether a single ethics approval covered the multi-country data collection or whether additional approvals and authorizations were required across partner institutions. The consent procedure should be described consistently and in sufficient detail to allow readers to understand how informed consent was obtained and managed at each site.

The Methods section would benefit from clearer terminology and a more transparent description of the analytical pipeline for mixed data dimensionality reduction and clustering. Currently, terms such as exploratory factor analysis of mixed data are presented alongside a workflow that includes PCA-based steps, hierarchical clustering, and K-means clustering, which can be confusing without precise specifications. The authors should clearly state which method was used to handle mixed data, for example, FAMD, MCA, or a specific PCA variant, and explain how categorical and ordinal variables were encoded, scaled, and weighted. The handling of missing data needs to be explicitly documented, including the amount of missingness, assumptions about the missingness mechanism, and whether imputation or listwise deletion was used to address missing data. The approach to outlier handling also requires stronger justification, including how many observations were excluded, why this decision was made, and whether key results remained stable when the analysis was repeated with and without these exclusions.

The rationale for selecting the number of clusters should be strengthened. A scree plot or Kaiser criterion is not typically used as a primary justification for the number of clusters, and cluster number selection should be supported by appropriate validation. The authors should include internal validation metrics, such as silhouette or gap statistics, and evaluate cluster stability using bootstrapping or split-sample validation. The manuscript should report whether the three-cluster solution remains robust under reasonable alternative analytical choices, including different seeds, distance measures, and clustering algorithms.

Another major concern is the sampling imbalance and nested structure of the data. The number of respondents varied substantially across universities, which can strongly influence the clustering outcomes and subsequent interpretation. The authors should present the cluster membership distributions by university and country and assess whether the clusters are driven by a small number of large sites. Sensitivity analyses using weighting or stratified resampling should be performed to ensure that the results are not dominated by a few high-response universities. When reporting associations between cluster membership and variables such as sex, field, or university, the nested nature of the data should be considered, at least through a clear discussion of the implications and ideally through multilevel or robust inference approaches.

Statistical reporting also requires careful attention. Several reported chi-square values appear as decimals, suggesting that some reported values may represent effect sizes, such as Cramér’s V, rather than chi-square test statistics. The authors should correct the labeling and report complete information for each test, including the test name, statistic, degrees of freedom, sample size, p-value, and effect size with interpretation. Tables should clearly indicate what each statistic represents to avoid misinterpretation of the results.

Finally, the interpretation should be more cautious and more tightly linked to the evidence. Cultural or regional explanations should not be overstated, particularly given the imbalance across universities and nested structure of the data. Broad East and West generalizations should be avoided unless supported by analyses that appropriately account for the site and country effects.

In terms of presentation, the manuscript would benefit from careful English editing to improve the clarity, grammar, and consistency of terminology. All variables used in the clustering should be clearly defined, including question wording, response scales, and coding decisions, either in the main text or in the supplementary materials. The interpretability of the clusters would be strengthened by adding a concise summary table that highlights the most discriminative variables for each cluster with effect sizes or other discriminative statistics and a brief practical interpretation of the table.

Reviewer #2: I would like to thank for the opportunity to review this manuscript. Please see the following comments to consider to further increase the quality of this manuscript.

This manuscript presents a large-scale, cross-national investigation of physical activity (PA) participation patterns among undergraduate students across 16 universities in 12 European countries. The study addresses a relevant public health issue—declining PA in emerging adulthood—and adopts an ecological perspective combined with cluster analysis of mixed data to identify typologies of engagement.

Comments:

The manuscript states that it adopts an ecological model (pp. 12–14), but the operationalization of this model is not sufficiently explicit. While determinants at individual, social, environmental, and policy levels are mentioned, the analysis primarily focuses on behavioral patterns and contextual variables.

The manuscript distinguishes between energetic definitions (Caspersen, 1985) and broader socio-cultural definitions (Piggin, 2020), yet the operationalization appears behaviorally categorical (e.g., supervised vs. autonomous; indoor vs. outdoor).

The sample is heavily skewed toward a few institutions (e.g., Prague: 24.1%; Rome & Teramo: 23.9%), while others contribute fewer than 3% of respondents (Table 2, pp. 16–17).

The manuscript notes that each university assisted in recruitment via institutional communication channels. This likely resulted in convenience sampling.

The two retained dimensions explain only 21.38% of total variance (p. 20). This is low for constructing typologies.

The manuscript states that a three-cluster solution was retained based on scree plot and Kaiser criterion (p. 19). However the Kaiser criterion is typically used in factor analysis, not clustering.

Outliers were excluded using the Grubb test (p. 20), which is typically designed for univariate normal data.

The claim that Eastern European students prefer more autonomous practice while Western/Southern students prefer organized practice (pp. 28–29) is intriguing but potentially overinterpreted.

The manuscript concludes that women are more represented in the “Autonomous” cluster and suggests potential discomfort in gyms (pp. 29–30). However:

“Use of ICT” is treated as a key profiling variable, but the construct is insufficiently described.

**********

what does this mean?). If published, this will include your full peer review and any attached files.

If you choose “no”, your identity will remain anonymous but your review may still be made public.

**Do you want your identity to be public for this peer review?**  For information about this choice, including consent withdrawal, please see our Privacy Policy

Reviewer #1: No

Reviewer #2: No

**********

[NOTE: If reviewer comments were submitted as an attachment file, they will be attached to this email and accessible via the submission site. Please log into your account, locate the manuscript record, and check for the action link "View Attachments". If this link does not appear, there are no attachment files.]

To ensure your figures meet our technical requirements, please review our figure guidelines: https://journals.plos.org/plosone/s/figures

You may also use PLOS’s free figure tool, NAAS, to help you prepare publication quality figures: https://journals.plos.org/plosone/s/figures#loc-tools-for-figure-preparation.

NAAS will assess whether your figures meet our technical requirements by comparing each figure against our figure specifications.

# Author comment: Author response to Decision Letter 1

**DOI:** 10.1371/journal.pone.0360751.r002

17 Jun 2026

Dear Professor Emily Chenette,

We sincerely thank you and the reviewers for carefully evaluating our manuscript and providing constructive and insightful comments. In response to the major points raised, we have thoroughly revised the manuscript and addressed each comment in detail.

In particular, we have substantially strengthened the methodological and statistical reporting. We clarified the analytical pipeline, including the use of factor analysis of mixed data, hierarchical clustering, and k-means consolidation. We revised the reporting of missing data, outlier handling, cluster validation, and statistical tests. We now report chi-square statistics, degrees of freedom, sample size, p-values, and Cramér’s V effect sizes more clearly.

Additionally, we carefully revised the interpretation of country, regional, and university-level patterns. To avoid overinterpretation, the manuscript no longer presents these findings as evidence of broad cultural or regional differences. Instead, they are now described as exploratory, institutional-level associations. Additional caution has been added in the Discussion and Limitations sections regarding the imbalance in recruitment and the nested structure of the data.

Several additional revisions were made to improve transparency and reproducibility. We updated the data availability statement, clarified the ethical reporting, and added supplementary materials, including variable definitions, coding information, explanatory notes for tables, a concise summary of discriminative variables by cluster, and internal validation indices for the cluster solution.

We revised the manuscript for English grammar, clarity, and consistency of terminology. All changes made in response to the reviewers' concerns are highlighted in the revised manuscript: blue for Reviewer 1, green for Reviewer 2, and orange for both.

We hope that these revisions adequately address the reviewers’ comments and improve the clarity, rigor, and reproducibility of the manuscript. We sincerely appreciate the opportunity to revise and resubmit our manuscript to PLOS ONE.

Yours sincerely,

The Authors

Attachment Submitted filename: PONE-D-25-68206_Response to Reviewers.docx

# Editor report: Decision Letter 1

**DOI:** 10.1371/journal.pone.0360751.r003

**Authors:** Alejandro Botero Carvajal

8 Jul 2026

PONE-D-25-68206R1

Where, When, How, and With Whom Do Young University Students Engage in Physical Activity? A Cluster-Derived Spectrum of Patterns Across 16 European Universities.

PLOS One

Dear Dr. Llena,

Thank you for submitting your manuscript to PLOS ONE. After careful consideration, we feel that it has merit but does not fully meet PLOS ONE’s publication criteria as it currently stands. Therefore, we invite you to submit a revised version of the manuscript that addresses the points raised during the review process.

================================

Please submit your revised manuscript by Aug 22 2026 11:59PM. If you will need more time than this to complete your revisions, please reply to this message or contact the journal office at plosone@plos.org. When you're ready to submit your revision, log on to https://www.editorialmanager.com/pone/ and select the 'Submissions Needing Revision' folder to locate your manuscript file.

- A letter that responds to each point raised by the academic editor and reviewer(s). You should upload this letter as a separate file labeled 'Response to Reviewers'.
- A marked-up copy of your manuscript that highlights changes made to the original version. You should upload this as a separate file labeled 'Revised Manuscript with Track Changes'.
- An unmarked version of your revised paper without tracked changes. You should upload this as a separate file labeled 'Manuscript'.

If you would like to make changes to your financial disclosure, please include your updated statement in your cover letter. Guidelines for resubmitting your figure files are available below the reviewer comments at the end of this letter.

If applicable, we recommend that you deposit your laboratory protocols in protocols.io to enhance the reproducibility of your results. Protocols.io assigns your protocol its own identifier (DOI) so that it can be cited independently in the future. For instructions see: https://journals.plos.org/plosone/s/submission-guidelines#loc-laboratory-protocols. Additionally, PLOS ONE offers an option for publishing peer-reviewed Lab Protocol articles, which describe protocols hosted on protocols.io. Read more information on sharing protocols at https://plos.org/protocols?utm_medium=editorial-email&utm_source=authorletters&utm_campaign=protocols.

As the corresponding author, your ORCID iD is verified in the submission system and will appear in the published article. PLOS supports the use of ORCID, and we encourage all coauthors to register for an ORCID iD and use it as well. Please encourage your coauthors to verify their ORCID iD within the submission system before final acceptance, as unverified ORCID iDs will not appear in the published article. *Only*  the individual author can complete the verification step; PLOS staff *cannot*  verify ORCID iDs on behalf of authors.

We look forward to receiving your revised manuscript.

Kind regards,

Alejandro Botero Carvajal, Ph.D

Academic Editor

PLOS One

Journal Requirements:

If the reviewer comments include a recommendation to cite specific previously published works, please review and evaluate these publications to determine whether they are relevant and should be cited. There is no requirement to cite these works unless the editor has indicated otherwise.

Please review your reference list to ensure that it is complete and correct. If you have cited papers that have been retracted, please include the rationale for doing so in the manuscript text, or remove these references and replace them with relevant current references. Any changes to the reference list should be mentioned in the rebuttal letter that accompanies your revised manuscript. If you need to cite a retracted article, indicate the article’s retracted status in the References list and also include a citation and full reference for the retraction notice.

[Note: HTML markup is below. Please do not edit.]

[NOTE: If reviewer comments were submitted as an attachment file, they will be attached to this email and accessible via the submission site. Please log into your account, locate the manuscript record, and check for the action link "View Attachments". If this link does not appear, there are no attachment files.]

To ensure your figures meet our technical requirements, please review our figure guidelines: https://journals.plos.org/plosone/s/figures

You may also use PLOS’s free figure tool, NAAS, to help you prepare publication quality figures: https://journals.plos.org/plosone/s/figures#loc-tools-for-figure-preparation.

NAAS will assess whether your figures meet our technical requirements by comparing each figure against our figure specifications.

# Author comment: Author response to Decision Letter 2

**DOI:** 10.1371/journal.pone.0360751.r004

13 Jul 2026

Dear Professor Emily Chenette,

Dears reviewers,

We sincerely thank you for your careful assessment of our revised manuscript. We are grateful for your positive evaluation of the substantial improvements made during the previous revision, as well as for recognizing that the manuscript is now technically sound and largely meets the scientific criteria of PLOS ONE.

In response to the remaining comments, we have carefully revised the manuscript and addressed each point in detail. We harmonized the Data Availability Statement across all submission files, which now clearly specifies the availability of the anonymized dataset, variable dictionary, and R analysis scripts in Recherche Data Gouv : https://doi.org/10.57745/HKG3H8.

Particular attention was given to moderating the interpretation of cultural and geographical differences. We reviewed the relevant sections of the abstract, introduction, discussion, limitations, and conclusion to ensure consistency throughout the manuscript. Statements suggesting broader cultural, geographical, national, or regional influences were removed or reformulated. The findings are now consistently described in terms of contextual characteristics and exploratory differences observed across participating institutions. We substantially revised the conclusion to ensure that the interpretation remains fully aligned with the study design and does not extend beyond what the data support.

Finally, we conducted a thorough review of the language and formatting. Any remaining grammatical, syntactic, or typographical errors were corrected, and the terminology was standardized throughout the manuscript and the supporting information.

All changes made are highlighted in purple in the revised manuscript.

We believe these revisions further improve the manuscript's clarity, consistency, and interpretive accuracy. We hope that the revised version fully addresses the remaining concerns and is suitable for publication in PLOS ONE.

Thank you again for the opportunity to revise and resubmit our work.

Yours sincerely,

The Authors

Date of resubmission: 11 July 2026

Attachment Submitted filename: Response to Reviewers_V2.docx

# Editor report: Decision Letter 2

**DOI:** 10.1371/journal.pone.0360751.r005

**Authors:** Alejandro Botero Carvajal

2 Aug 2026

PONE-D-25-68206R2

Where, When, How, and With Whom Do Young University Students Engage in Physical Activity? A Cluster-Derived Spectrum of Patterns Across 16 European Universities.

PLOS One?

Dear Dr. Llena,

Thank you for submitting your manuscript to PLOS ONE. After careful consideration, we feel that it has merit but does not fully meet PLOS ONE’s publication criteria as it currently stands. Therefore, we invite you to submit a revised version of the manuscript that addresses the points raised during the review process.

Please submit your revised manuscript by Sep 14 2026 11:59PM. If you will need more time than this to complete your revisions, please reply to this message or contact the journal office at plosone@plos.org. When you're ready to submit your revision, log on to https://www.editorialmanager.com/pone/ and select the 'Submissions Needing Revision' folder to locate your manuscript file.

- A letter that responds to each point raised by the academic editor and reviewer(s). You should upload this letter as a separate file labeled 'Response to Reviewers'.
- A marked-up copy of your manuscript that highlights changes made to the original version. You should upload this as a separate file labeled 'Revised Manuscript with Track Changes'.
- An unmarked version of your revised paper without tracked changes. You should upload this as a separate file labeled 'Manuscript'.

If you would like to make changes to your financial disclosure, please include your updated statement in your cover letter. Guidelines for resubmitting your figure files are available below the reviewer comments at the end of this letter.

If applicable, we recommend that you deposit your laboratory protocols in protocols.io to enhance the reproducibility of your results. Protocols.io assigns your protocol its own identifier (DOI) so that it can be cited independently in the future. For instructions see: https://journals.plos.org/plosone/s/submission-guidelines#loc-laboratory-protocols. Additionally, PLOS ONE offers an option for publishing peer-reviewed Lab Protocol articles, which describe protocols hosted on protocols.io. Read more information on sharing protocols at https://plos.org/protocols?utm_medium=editorial-email&utm_source=authorletters&utm_campaign=protocols.

As the corresponding author, your ORCID iD is verified in the submission system and will appear in the published article. PLOS supports the use of ORCID, and we encourage all coauthors to register for an ORCID iD and use it as well. Please encourage your coauthors to verify their ORCID iD within the submission system before final acceptance, as unverified ORCID iDs will not appear in the published article. *Only*  the individual author can complete the verification step; PLOS staff *cannot*  verify ORCID iDs on behalf of authors.

We look forward to receiving your revised manuscript.

Kind regards,

Alejandro Botero Carvajal, Ph.D

Academic Editor

PLOS One

Journal Requirements:

If the reviewer comments include a recommendation to cite specific previously published works, please review and evaluate these publications to determine whether they are relevant and should be cited. There is no requirement to cite these works unless the editor has indicated otherwise.

Please review your reference list to ensure that it is complete and correct. If you have cited papers that have been retracted, please include the rationale for doing so in the manuscript text, or remove these references and replace them with relevant current references. Any changes to the reference list should be mentioned in the rebuttal letter that accompanies your revised manuscript. If you need to cite a retracted article, indicate the article’s retracted status in the References list and also include a citation and full reference for the retraction notice.

Additional Editor Comments:

Dear Authors,

Thank you for submitting the revised version of your manuscript. The manuscript has improved substantially, and the principal scientific and methodological concerns raised in the previous rounds have now been addressed. In particular, the analytical workflow is more transparent, cluster validation is reported more clearly, FAMD terminology is used consistently, the interpretation of institutional differences has been moderated, and the data dictionary, anonymised dataset, and R analysis scripts have been assigned a repository DOI. The manuscript is therefore close to being suitable for publication. Before a final decision can be made, please address the following remaining points:

Data Availability Statement: The clean title page still states that “All relevant data are within the manuscript,” whereas the main manuscript and submission metadata identify the Recherche Data Gouv repository. Please replace the title-page statement with the harmonised repository statement and verify that the DOI provides access to the anonymised dataset, data dictionary, and R scripts in accordance with PLOS ONE policy.

Participant flow: Please provide a transparent breakdown of exclusions from the 4,321 initial respondents to the 2,165 participants included in the final analysis. Report the number excluded for each eligibility criterion, incomplete data, inconsistent responses, health-related exclusions, and outlier screening. Where feasible, provide a brief comparison of included and excluded respondents or explain why this could not be performed.

Interpretation of sex-related findings: Please remove or substantially qualify the recommendation for women-only spaces. The study did not assess perceived safety, comfort, belonging, or the effectiveness of sex-specific interventions, and the observed association between sex and cluster membership was small. Such proposals should be presented as questions for future research rather than evidence-based recommendations from the present study.

Intervention and effectiveness language: Please revise statements suggesting that particular PA formats are “effective,” will “enhance” activity levels, or will produce healthier routines. The cross-sectional exploratory design identifies participation profiles and associations but does not evaluate intervention effectiveness or changes in PA. Implications should be framed as hypotheses or considerations for future prospective and intervention research.

Ethics statement: Please ensure that the ethics statement appears only in the Methods section, in accordance with the journal’s previous instruction, and remove the redundant statement from the title page if required.

Final consistency check: Please perform one final proofread to ensure that cluster labels, percentages, institutional names, funding statements, and supplementary-file references are identical across the clean manuscript, title page, tracked version, response letter, and submission metadata.

These are limited but mandatory corrections. Provided that they are addressed satisfactorily, the manuscript should be suitable for acceptance without a further round of external peer review.

Sincerely,

Academic Editor

PLOS ONE

[Note: HTML markup is below. Please do not edit.]

[NOTE: If reviewer comments were submitted as an attachment file, they will be attached to this email and accessible via the submission site. Please log into your account, locate the manuscript record, and check for the action link "View Attachments". If this link does not appear, there are no attachment files.]

To ensure your figures meet our technical requirements, please review our figure guidelines: https://journals.plos.org/plosone/s/figures

You may also use PLOS’s free figure tool, NAAS, to help you prepare publication quality figures: https://journals.plos.org/plosone/s/figures#loc-tools-for-figure-preparation.

NAAS will assess whether your figures meet our technical requirements by comparing each figure against our figure specifications.

# Author comment: Author response to Decision Letter 3

**DOI:** 10.1371/journal.pone.0360751.r006

5 Aug 2026

Dear Professor Alejandro Botero Carvajal,

Thank you again for the opportunity to revise and resubmit our work. We have carefully addressed all of the mandatory corrections you identified in your editorial assessment. A detailed, point-by-point response is provided in the separate Response to Reviewers document.

The main outstanding issue concerned data availability. We have deposited the anonymized dataset, variable dictionary, and R analysis script in Recherche Data Gouv under the DOI 10.57745/HKG3H8. The dataset and variable dictionary have been validated and are available through the repository. The R analysis script was also deposited under the same DOI, but it is still awaiting validation by the University of Lille and the repository platform. We are following up with the repository team to ensure it is publicly accessible before publication. The manuscript now contains a harmonized data availability statement referring to all three deposited materials. We have also attached confirmation that the R analysis script was submitted to the repository.

We revised the manuscript to clarify participant flow, qualify the interpretation of the sex-related findings, remove unsupported claims about the effectiveness of the intervention, retain the ethics statement only in the Methods section, and ensure consistency across all submission files. We reviewed the complete reference list and did not identify any retracted articles.

We hope that the revised manuscript and response address all remaining editorial requirements.

Yours sincerely,

The Authors

Attachment Submitted filename: Response to Reviewers_V3.docx

# Editor report: Decision Letter 3

**DOI:** 10.1371/journal.pone.0360751.r007

**Authors:** Alejandro Botero Carvajal

16 Aug 2026

PONE-D-25-68206R3

Where, When, How, and With Whom Do Young University Students Engage in Physical Activity? A Cluster-Derived Spectrum of Patterns Across 16 European Universities.

PLOS One?

Dear Dr. Llena,

Thank you for submitting your manuscript to PLOS ONE. After careful consideration, we feel that it has merit but does not fully meet PLOS ONE’s publication criteria as it currently stands. Therefore, we invite you to submit a revised version of the manuscript that addresses the points raised during the review process.

Please submit your revised manuscript by Oct 14 2026 11:59PM. If you will need more time than this to complete your revisions, please reply to this message or contact the journal office at plosone@plos.org. When you're ready to submit your revision, log on to https://www.editorialmanager.com/pone/ and select the 'Submissions Needing Revision' folder to locate your manuscript file.

- A letter that responds to each point raised by the academic editor and reviewer(s). You should upload this letter as a separate file labeled 'Response to Reviewers'.
- A marked-up copy of your manuscript that highlights changes made to the original version. You should upload this as a separate file labeled 'Revised Manuscript with Track Changes'.
- An unmarked version of your revised paper without tracked changes. You should upload this as a separate file labeled 'Manuscript'.

If you would like to make changes to your financial disclosure, please include your updated statement in your cover letter. Guidelines for resubmitting your figure files are available below the reviewer comments at the end of this letter.

If applicable, we recommend that you deposit your laboratory protocols in protocols.io to enhance the reproducibility of your results. Protocols.io assigns your protocol its own identifier (DOI) so that it can be cited independently in the future. For instructions see: https://journals.plos.org/plosone/s/submission-guidelines#loc-laboratory-protocols. Additionally, PLOS ONE offers an option for publishing peer-reviewed Lab Protocol articles, which describe protocols hosted on protocols.io. Read more information on sharing protocols at https://plos.org/protocols?utm_medium=editorial-email&utm_source=authorletters&utm_campaign=protocols.

As the corresponding author, your ORCID iD is verified in the submission system and will appear in the published article. PLOS supports the use of ORCID, and we encourage all coauthors to register for an ORCID iD and use it as well. Please encourage your coauthors to verify their ORCID iD within the submission system before final acceptance, as unverified ORCID iDs will not appear in the published article. *Only*  the individual author can complete the verification step; PLOS staff *cannot*  verify ORCID iDs on behalf of authors.

We look forward to receiving your revised manuscript.

Kind regards,

Alejandro Botero Carvajal, Ph.D

Academic Editor

PLOS One

Journal Requirements:

If the reviewer comments include a recommendation to cite specific previously published works, please review and evaluate these publications to determine whether they are relevant and should be cited. There is no requirement to cite these works unless the editor has indicated otherwise.

Please review your reference list to ensure that it is complete and correct. If you have cited papers that have been retracted, please include the rationale for doing so in the manuscript text, or remove these references and replace them with relevant current references. Any changes to the reference list should be mentioned in the rebuttal letter that accompanies your revised manuscript. If you need to cite a retracted article, indicate the article’s retracted status in the References list and also include a citation and full reference for the retraction notice.

Additional Editor Comments :

Decision: Minor Revision

The manuscript has improved substantially, and the authors have adequately addressed the major scientific and interpretative concerns raised in the previous editorial assessment. The participant flow is now transparent, the limitations associated with convenience sampling and unequal institutional representation are appropriately acknowledged, and causal, intervention-effectiveness, and sex-specific recommendations have been satisfactorily qualified. The analytical approach and exploratory interpretation of the cluster profiles are now generally suitable for publication.

Before the manuscript can be accepted, a small number of mandatory issues must be resolved. First, the R analysis script must be publicly accessible through the stated repository DOI, or the Data Availability Statement must accurately reflect its current accessibility. Second, the denominators used for the percentages in Table 4 must be explicitly defined, particularly because several displayed rows do not sum to 100% across clusters. Third, the manuscript must clarify whether the sample comprised exclusively first-year students or undergraduate students from multiple academic years and use consistent terminology throughout. Finally, the statement that participation format and regularity differentiate students more strongly than quantity or intensity should be moderated to reflect the exploratory nature of the analysis.

These issues do not require a new external review and may be assessed editorially. Subject to satisfactory correction and verification of public access to the underlying data, dictionary, and analysis code, the manuscript would be suitable for acceptance.

[Note: HTML markup is below. Please do not edit.]

[NOTE: If reviewer comments were submitted as an attachment file, they will be attached to this email and accessible via the submission site. Please log into your account, locate the manuscript record, and check for the action link "View Attachments". If this link does not appear, there are no attachment files.]

To ensure your figures meet our technical requirements, please review our figure guidelines: https://journals.plos.org/plosone/s/figures

You may also use PLOS’s free figure tool, NAAS, to help you prepare publication quality figures: https://journals.plos.org/plosone/s/figures#loc-tools-for-figure-preparation.

NAAS will assess whether your figures meet our technical requirements by comparing each figure against our figure specifications.

# Author comment: Author response to Decision Letter 4

**DOI:** 10.1371/journal.pone.0360751.r008

19 Aug 2026

Dear Mrs Maderazo,

Thank you for highlighting this point,

Following your latest comment, we have now also revised the Data Availability Statement to explicitly identify Figshare as the repository. The updated statement has been included in the resubmitted manuscript.

Yours sincerely,

The Authors

Date of resubmission: 19 August 2026

Attachment Submitted filename: Response to Reviewers_V4.docx

# Editor report: Decision Letter 4

**DOI:** 10.1371/journal.pone.0360751.r009

**Authors:** Alejandro Botero Carvajal

7 Sep 2026

PONE-D-25-68206R4

Where, When, How, and With Whom Do Young University Students Engage in Physical Activity? A Cluster-Derived Spectrum of Patterns Across 16 European Universities.

PLOS One?

Dear Dr. Llena,

Thank you for submitting your manuscript to PLOS One. After careful consideration, we feel that it has merit but does not fully meet PLOS One’s publication criteria as it currently stands. Therefore, we invite you to submit a revised version of the manuscript that addresses the points raised during the review process.

Please submit your revised manuscript by Oct 22 2026 11:59PM. If you will need more time than this to complete your revisions, please reply to this message or contact the journal office at plosone@plos.org. When you're ready to submit your revision, log on to https://www.editorialmanager.com/pone/ and select the 'Submissions Needing Revision' folder to locate your manuscript file.

- A letter that responds to each point raised by the academic editor and reviewer(s). You should upload this letter as a separate file labeled 'Response to Reviewers'.
- A marked-up copy of your manuscript that highlights changes made to the original version. You should upload this as a separate file labeled 'Revised Manuscript with Track Changes'.
- An unmarked version of your revised paper without tracked changes. You should upload this as a separate file labeled 'Manuscript'.

If you would like to make changes to your financial disclosure, please include your updated statement in your cover letter. Guidelines for resubmitting your figure files are available below the reviewer comments at the end of this letter.

If applicable, we recommend that you deposit your laboratory protocols in protocols.io to enhance the reproducibility of your results. Protocols.io assigns your protocol its own identifier (DOI) so that it can be cited independently in the future. For instructions see: https://journals.plos.org/plosone/s/submission-guidelines#loc-laboratory-protocols. Additionally, PLOS One offers an option for publishing peer-reviewed Lab Protocol articles, which describe protocols hosted on protocols.io. Read more information on sharing protocols at https://plos.org/protocols?utm_medium=editorial-email&utm_source=authorletters&utm_campaign=protocols.

As the corresponding author, your ORCID iD is verified in the submission system and will appear in the published article. PLOS supports the use of ORCID, and we encourage all coauthors to register for an ORCID iD and use it as well. Please encourage your coauthors to verify their ORCID iD within the submission system before final acceptance, as unverified ORCID iDs will not appear in the published article. *Only*  the individual author can complete the verification step; PLOS staff *cannot*  verify ORCID iDs on behalf of authors.

We look forward to receiving your revised manuscript.

Kind regards,

Alejandro Botero Carvajal, Ph.D

Academic Editor

PLOS One

Journal Requirements:

If the reviewer comments include a recommendation to cite specific previously published works, please review and evaluate these publications to determine whether they are relevant and should be cited. There is no requirement to cite these works unless the editor has indicated otherwise.

Please review your reference list to ensure that it is complete and correct. If you have cited papers that have been retracted, please include the rationale for doing so in the manuscript text, or remove these references and replace them with relevant current references. Any changes to the reference list should be mentioned in the rebuttal letter that accompanies your revised manuscript. If you need to cite a retracted article, indicate the article’s retracted status in the References list and also include a citation and full reference for the retraction notice.

Additional Editor Comments (if provided):

The authors have satisfactorily addressed the substantive editorial concerns. The manuscript is suitable for acceptance, subject to correction of one numerical inconsistency: the combined proportion of the two supervised clusters is 57.4% rather than 67.4%. Please also ensure that the age range is reported consistently throughout the manuscript.

[Note: HTML markup is below. Please do not edit.]

[NOTE: If reviewer comments were submitted as an attachment file, they will be attached to this email and accessible via the submission site. Please log into your account, locate the manuscript record, and check for the action link "View Attachments". If this link does not appear, there are no attachment files.]

To ensure your figures meet our technical requirements, please review our figure guidelines: https://journals.plos.org/plosone/s/figures

You may also use PLOS’s free figure tool, NAAS, to help you prepare publication quality figures: https://journals.plos.org/plosone/s/figures#loc-tools-for-figure-preparation.

NAAS will assess whether your figures meet our technical requirements by comparing each figure against our figure specifications.

# Author comment: Author response to Decision Letter 5

**DOI:** 10.1371/journal.pone.0360751.r010

11 Sep 2026

Thank you for pointing this out.

We have carefully reviewed the submission file inventory and removed the duplicated and outdated files.

Kind regards,

Attachment Submitted filename: Response to Reviewers_V5.docx

# Editor report: Decision Letter 5

**DOI:** 10.1371/journal.pone.0360751.r011

**Authors:** Alejandro Botero Carvajal

30 Sep 2026

Where, When, How, and With Whom Do Young University Students Engage in Physical Activity? A Cluster-Derived Spectrum of Patterns Across 16 European Universities.

PONE-D-25-68206R5

Dear Dr. Llena,

We’re pleased to inform you that your manuscript has been judged scientifically suitable for publication and will be formally accepted for publication once it meets all outstanding technical requirements.

Within one week, you’ll receive an e-mail detailing the required amendments. When these have been addressed, you’ll receive a formal acceptance letter and your manuscript will be scheduled for publication.

An invoice will be generated when your article is formally accepted. Please note, if your institution has a publishing partnership with PLOS and your article meets the relevant criteria, all or part of your publication costs will be covered. Please make sure your user information is up-to-date by logging into Editorial Manager at Editorial Manager® and clicking the ‘Update My Information' link at the top of the page. For questions related to billing, please contact billing support.

If your institution or institutions have a press office, please notify them about your upcoming paper to help maximize its impact. If they’ll be preparing press materials, please inform our press team as soon as possible -- no later than 48 hours after receiving the formal acceptance. Your manuscript will remain under strict press embargo until 2 pm Eastern Time on the date of publication. For more information, please contact onepress@plos.org.

Kind regards,

Alejandro Botero Carvajal, Ph.D

Academic Editor

PLOS One

Additional Editor Comments (optional):

Reviewers' comments:

# Editor report: Acceptance letter

**DOI:** 10.1371/journal.pone.0360751.r012

**Authors:** Alejandro Botero Carvajal

PONE-D-25-68206R5

PLOS One

Dear Dr. Llena,

I'm pleased to inform you that your manuscript has been deemed suitable for publication in PLOS One. Congratulations! Your manuscript is now being handed over to our production team.

At this stage, our production department will prepare your paper for publication. This includes ensuring the following:

* All references, tables, and figures are properly cited

* All relevant supporting information is included in the manuscript submission,

* There are no issues that prevent the paper from being properly typeset

You will receive further instructions from the production team, including instructions on how to review your proof when it is ready. Please keep in mind that we are working through a large volume of accepted articles, so please give us a few days to review your paper and let you know the next and final steps.

Lastly, if your institution or institutions have a press office, please let them know about your upcoming paper now to help maximize its impact. If they'll be preparing press materials, please inform our press team within the next 48 hours. Your manuscript will remain under strict press embargo until 2 pm Eastern Time on the date of publication. For more information, please contact onepress@plos.org.

You will receive an invoice from PLOS for your publication fee after your manuscript has reached the completed accept phase. If you receive an email requesting payment before acceptance or for any other service, this may be a phishing scheme. Learn how to identify phishing emails and protect your accounts at https://explore.plos.org/phishing.

If we can help with anything else, please email us at customercare@plos.org.

Thank you for submitting your work to PLOS One and supporting open access.

Kind regards,

PLOS One Editorial Office Staff

on behalf of

Dr. Alejandro Botero Carvajal

Academic Editor

PLOS One
